# An intermediate activation state primes Langerhans cell migration from the epidermis

**DOI:** 10.1101/2025.05.29.656912

**Published:** 2025-05-30

**Authors:** Artem Kiselev, Axel D. Schmitter-Sánchez, Sharod Williams, Sangbum Park

**Affiliations:** 1 Institute for Quantitative Health Science and Engineering (IQ), Michigan State University, East Lansing, MI, USA.; 2 Cell and Molecular Biology Program, College of Natural Science, Michigan State University, East Lansing, MI, USA; 3 RTSF Genomics Core, Michigan State University, East Lansing, MI, USA.; 4 Division of Dermatology, Department of Medicine, College of Human Medicine, Michigan State University, East Lansing, MI, USA.; 5 Department of Pharmacology and Toxicology, College of Human Medicine, Michigan State University, East Lansing, MI, USA.

**Keywords:** Langerhans cells, Single-Cell RNA Sequencing, Activation, Wound, Complement system

## Abstract

Langerhans cells (LCs) are a specialized subset of dendritic cells residing in the epidermis, where they form a dense network that functions as a frontline defense by detecting environmental antigens and sensing skin damage. Despite extensive research, many immunological and physiological aspects of LCs function remain poorly understood. In this study, we combined single-cell transcriptomic analysis and intravital imaging to reconstruct the activation trajectory of epidermal LCs and identified a distinct intermediate state that precedes their migration. We present a high-resolution single-cell transcriptomic dataset of over 22,000 high-quality epidermal LCs under both injured and homeostatic conditions. We found similar LCs responses to chemical and physical skin injuries. Our analysis defines specific LCs subpopulations representing sequential activation stages, characterized at the level of pathways and transcription factors. Notably, we identified a previously unrecognized population of activated LCs distinguished by the expression of complement system components and receptors. Integrating our data with an external dataset of wounded and unwounded skin revealed that wound-specific, WNT-modulated fibroblasts are the primary source of C3, the central component of the complement cascade. Intravital imaging of C3-deficient mice demonstrated that C3 is essential for the effective recruitment of activated LCs to wound sites. Together, these findings uncover a novel population of activated epidermal LCs and highlight complement signaling as a critical mediator of LCs recruitment during skin injury.

## Introduction

Langerhans cells (LCs) are a specialized subset of dendritic cells that reside in the epidermis^1^, where they form a dense network and function as immune sentinels by detecting environmental antigens, sensing tissue damage, and initiating immune responses and antigen delivery to lymph node^2^. LCs have been extensively studied as antigen-presenting cells (APC), and their development, differentiation, and immunological roles have been well documented. However, it remains unclear how LCs undergo transcriptomic changes during activation and what functional consequences these changes have in vivo^3–5^.

To address this, we combined high-resolution single-cell RNA sequencing (scRNA-seq) with intravital imaging to reconstruct the activation trajectory of epidermal LCs under homeostatic and injury conditions, providing novel insights into their dynamic behavior and functional states in the native tissue environment.

Recent advancements in scRNA-seq have significantly enhanced our understanding of LCs biology, revealing previously unrecognized heterogeneity and functional specialization within this cell type^6,7^. For instance, a study by Kim et al. (2024) identified four distinct LCs subpopulations in human skin: two steady state and two pro-inflammatory/matured subsets. Notably, the pro-inflammatory subsets were enriched in atopic dermatitis lesions, suggesting a role in disease pathogenesis^8^. Further, spatial and single-cell transcriptomic analyses have delineated the intercellular communication networks between LCs and other skin-resident cells, such as keratinocytes and fibroblasts. These studies have uncovered how LCs interact with their microenvironment to maintain skin homeostasis and respond to inflammatory stimuli^9^. In murine models, scRNA-seq has revealed that LCs exhibit a dual identity, functioning both as tissue-resident macrophages and as migrating antigen-presenting cells. This duality is influenced by intrinsic factors and the epidermal niche, highlighting the complexity of LCs ontogeny and function^9^. scRNA-seq have revolutionized our ability to dissect LCs cellular landscapes, enabling high-resolution profiling of individual LCs populations within epidermis^10^. From other hand, intravital imaging, combined with genetic modification and fluorescent protein labeling, offers powerful opportunities for real-time monitoring of cells in vivo. This is particularly valuable for studying LCs, one of the few immune cells whose behavior can be observed in an intact organism and directly compared under different conditions and treatments by imaging analysis.

In this study, we integrated single-cell RNA sequencing, intravital microscopy, and refined experimental protocols to enable large-scale activation and rapid isolation of viable LCs. Our optimized workflow ensured high purity and viability, supporting deep transcriptional profiling of LCs functional states. We identified distinct LCs subsets with unique gene expression signatures, uncovering functional diversification within the injured epidermis. Notably, we characterized for the discrete LCs populations in both homeostatic and injured skin. These insights advance our understanding of LCs biology and establish a valuable framework for future research into skin immunity and disease.

## Results

### Physical and chemical treatment causes similar LCs activation trajectory

In non-inflamed skin LCs form a tight network across the epidermis, with each cell maintaining relatively equal spacing^11,12^. This organized distribution supports efficient immune surveillance and is sustained through local proliferation of resident LCs under homeostatic conditions. However, under skin insults, the LCs loses regular spatial distribution. This disorganization likely facilitates the acquisition of antigens by enabling LCs to access damaged areas more freely, where they can capture environmental or endogenous antigens before migrating to the draining lymph node to initiate adaptive immune responses^13^.

**Fig. 1.**
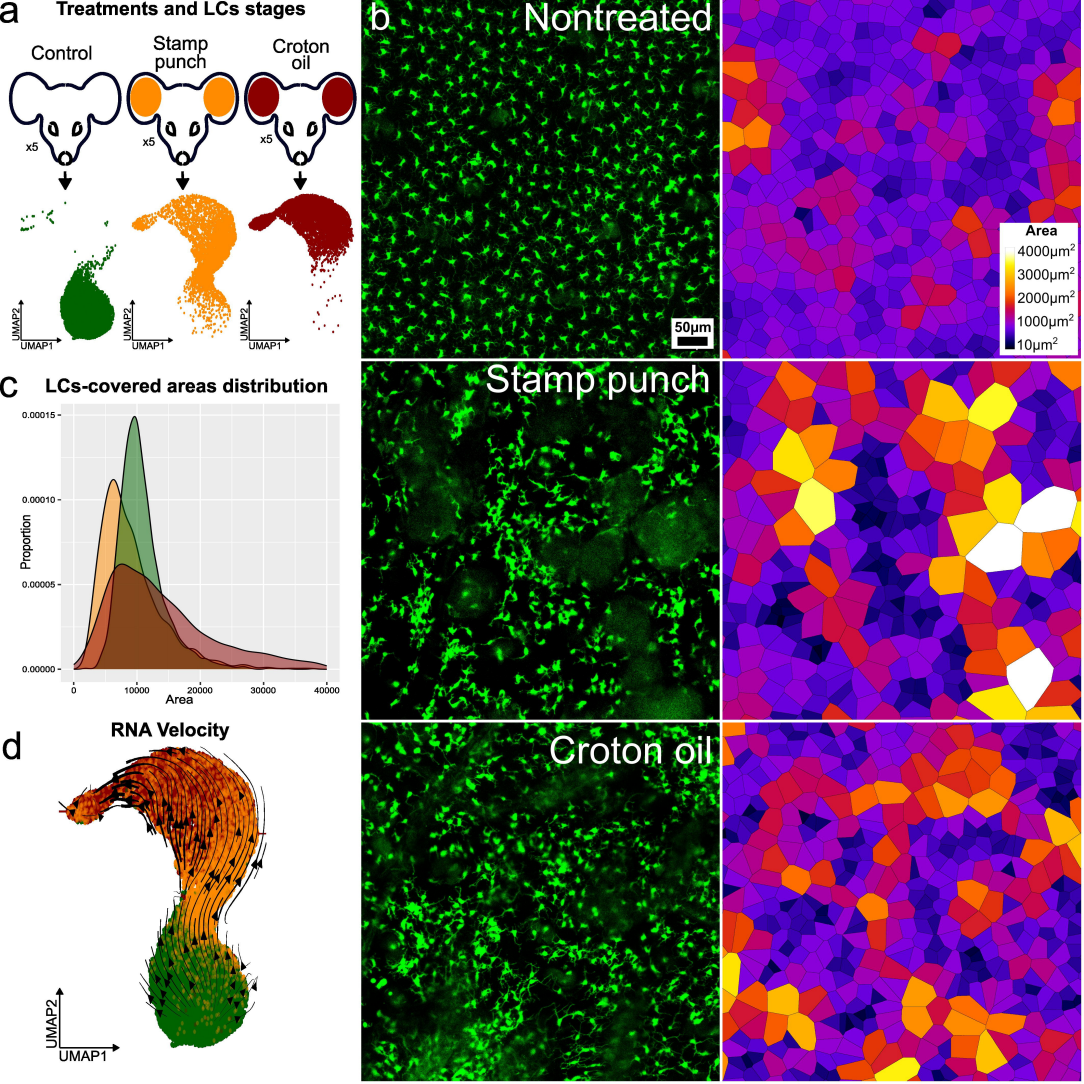
Experimental design and in vivo dynamics of LCs activation and migration. a Schematic overview of the experimental design for single-cell RNA sequencing (scRNA-seq) data acquisition; b Intravital imaging of mouse ear epidermis under three conditions: untreated control, 48 hours post-croton oil treatment, and 48 hours post-stamp-punch stimulation. Corresponding Voronoi diagrams illustrate changes in LCs organization; c Distribution of cell territories derived from Voronoi diagram analysis highlights significant spatial differences between control and treated samples; c RNA velocity analysis reveals the directional trajectory of LCs, supporting the sequential progression from steady state to activated and migratory states.

To define LCs behavior during injury, we utilized intravital microscopy in mice expressing GFP under the control of the Cd207 (Langerin) promoter^14^. We established two effective skin injury approaches and identified optimal time points at which the disruption of the LCs network was most efficient, while the majority of LCs remained within the epidermis. The first method, classified as physical, involved repeated puncturing of mouse ear skin using a 0.2 mm microneedle array, referred to as the stamp-punch. (Fig. 1 a b). The second method, classified as chemical, involved a 10-minute treatment of the ear skin with a mixture of croton oil^15^, acetone, and olive oil, referred to as the croton/acetone. In both the physical and chemical treatments, LCs activation peaked around 46 hours post-treatment, with disrupted spatial patterns but a total cell number comparable to the homeostatic state (Fig. 1 b c).

For scRNA-seq using the 10x Genomics platform, we isolated LCs from three experimental groups: two corresponding to the activation methods described above and one untreated control group. Each scRNA-seq sample consisted of pooled epidermal LCs extracted from the ear skin of five mice per group. Single-cell transcriptomic analysis revealed that LCs from the stamp-punch and croton/acetone-treated groups, together with the control, formed a continuous trajectory reflecting progressive activation and migration. To validate this findings, we applied RNA velocity^16^ analysis. This approach confirmed that, in response to both skin treatment and injury, LCs first transition into a distinct activated state before progressing to a migratory state (Fig. 1 d). The trajectory appeared to be unidirectional and irreversible over the 46-hour observation period, regardless of whether the stimulus was physical or chemical. This pattern was consistent across all three experimental groups, indicating a single, shared activation program. Differences were observed only in the extent of activation, reflected by varying enrichment in specific experimental group.

Using intravital imaging, we identified the 46-hour time point as optimal for LCs isolation, as the network is fully disrupted while a substantial number of cells still remain in the injured epidermis. scRNA-seq profiling of LCs isolated 46 hours after injury revealed that both physical and chemical stimuli induce a shared activation trajectory.

### Identification of an intermediate state of LCs preceding epidermal exit

**Fig. 2.**
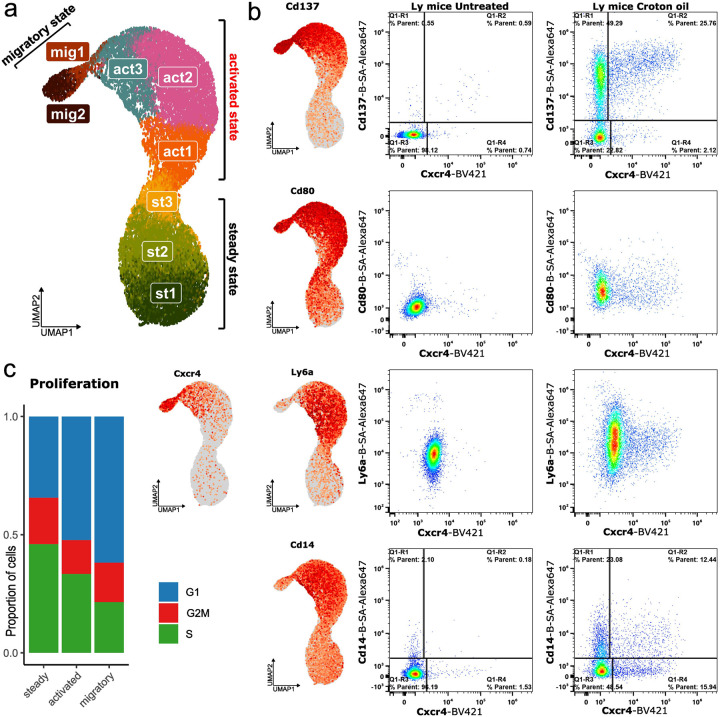
Transcriptional and functional characterization of LCs activation state. a scRNA-seq analysis identified three major transcriptional states of LCs —steady, activated, and migratory — separated by UMAP clustering. For in-depth regulon and pathway analysis, the dataset was further subdivided into eight transcriptionally distinct substages; b Differential gene expression and flow cytometry analysis demonstrated that activated LCs can be distinguished from steady and migratory populations using surface markers such as Cd137, Cd14, and Ly6a; c During activation, the majority of LCs exit the proliferative cycle and undergo mitotic arrest, indicating a functional shift from self-renewal to immune engagement.

Basic integration of samples and data analysis revealed three distinct clusters on UMAP (Fig. 2 a). We annotated them as steady, migratory and intermediate activated clusters or states. Steady state LCs, representing quiescent cells residing in healthy/untreated epidermis, had the high level of expression of *Cd207*^17^, as well as base low levels of *Cd80*^18^ and *Cd86*^19^ markers, associated with the activation of antigen presenting cells. The migratory state, identified by high expression of *Cxcr4*^20^ and *Ccr7*^21^, formed the smallest cluster of cells that have almost left the epidermis, representing approximately 5% of the dataset (Fig 2 b). Between steady and migratory populations we identified an intermediate activated LCs population, a transitional LCs state that has received limited attention in the existing literature^7,22,23^.

As the next step, we aimed to further characterize the identified LCs populations by identifying specific markers for each state, with a particular focus on the activated population. We combined bioinformatic analysis with flow cytometry-based protein-level validation. Using differential expression (DE) analysis we identify unique markers of each LCs population as well as shared features among groups. Our analysis identified approximately 400 transcripts that clearly separated the activated state LCs from the steady and/or the migratory states (Supplementary Table 1). Among the identified transcripts, one of the initial findings was the upregulation of well-established antigen-presenting cell activation markers, such as *Cd80* and *Cd86*, during both the activated and migratory states (Fig 2 b). However, analysis of both transcriptomic data and flow cytometry results showed that these markers also exhibited relatively high baseline expression in the steady state LCs, limiting clear discrimination between the activated and migratory states from steady state. To address this limitation, we prioritized the identification of alternative markers that more precisely delineate the transitions between steady, activated, and migratory states at both the transcriptomic and protein levels. From the transcripts set specific to the activation state, we shortlisted 12 candidate genes based on predicted cell surface localization or roles as transcription factors. The chosen transcripts included *Cd14*^24^, *Ly6a*^25^, *Il1b*, *Cd137* (*Tnfrsf9*)^26^, Ccr*7* (*Cd197), Cd49d* (*Itga4*), *Cd274*^27,28^, *Cxcr4*, *Adgre4* (*Emr4*), *Mafb*^29^, and *Bhlhe40*^30^. We focused specifically on transcripts with low or undetectable expression in one or more states, prioritizing those that either showed a progressive increase from the steady to migratory state or were selectively expressed in a single state with minimal expression in others. To validate these findings at the protein level, we performed flow cytometry analysis using antibodies corresponding to each of the selected markers (Fig 2 b). The most pronounced separation of LCs populations was achieved using a combination of surface markers, including the previously unreported LCs activation markers *Cd137*, *Ly6a*, and *Cd14*, along with the known migratory marker *Cxcr4* (Fig 2 b). Among these, the combination of biotinylated *Cd137* and fluorochrome-conjugated *Cxcr4* provided the clearest distinction of the activated state LCs population (Fig 2 b). In this setup, *Cd137*-negative cells corresponded to steady state LCs, while *Cd137*^+^ cells represented activated state LCs transitioning toward the migratory state. Additional markers, *Cd14* and *Ly6a*, also exhibited state specific expression patterns, peaking in the activated state and subsequently declining as cells advanced toward migration state (Fig 2 b).

Altogether, through integrated bioinformatic analysis and flow cytometry, we delineated three transcriptionally and phenotypically distinct states of LCs during activation in the epidermis. Notably, we identified *Cd137* and *Ly6a* as specific surface markers enriched in the intermediate activated state, providing precise identification and future functional characterization. In addition, we found that during the transition to the activation and migration states, LCs proliferation activity decreases and most LCs enter the G1 arrest^31^ stage of mitosis (Fig 2 c).

### Transcriptional and functional reprogramming of LCs activation

**Fig. 3.**
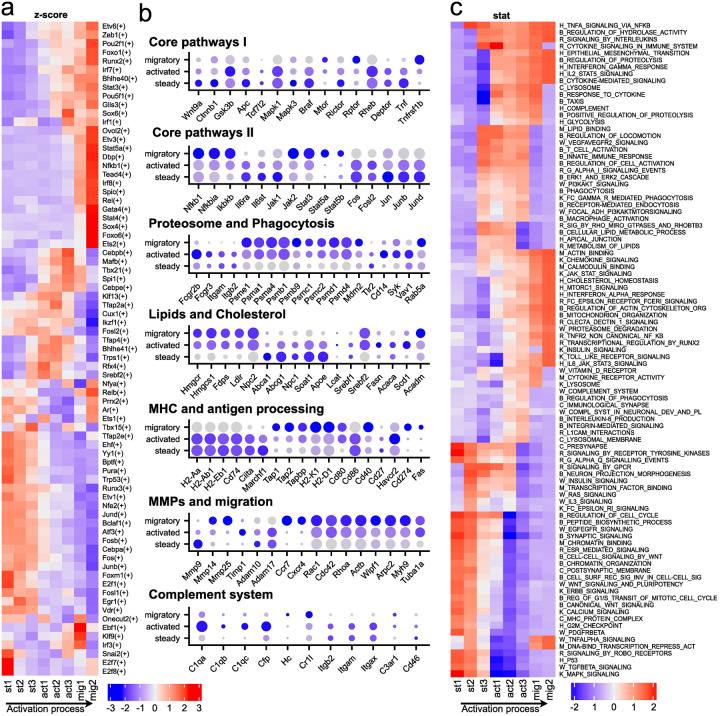
Regulatory network and pathway dynamics underlying LCs activation and migration. a Heatmap generated using the SCENIC tool, where red indicates high activity and blue indicates low activity by z-score of gene regulatory networks associated with specific transcription factors; b Dot plots showing the relative expression of key genes during the activation and migration of LCs. Dot size reflects the proportion of cells expressing the gene, while color intensity represents expression levels; c Pathway enrichment analysis using the SCPA package. Red indicates high pathway activity, and blue indicates low activity. Prefixes before pathway names denote the source database: H – Hallmarks, B – GO: Biological Processes, M – GO: Molecular Functions, C – GO: Cellular Components, R – Reactome, W – WikiPathways, and K – KEGG.

To uncover the key regulatory mechanisms driving transitions between LCs states, we explored transcription factor pathways and networks. We subdivided the three defined states into eight transcriptionally distinct substates. We then applied transcription factor network activity analysis using SCENIC and assessed regulatory signaling dynamics through Single-Cell Pathway Analysis (SCPA), enabling us to track coordinated pathway changes across the LCs activation continuum. Both of these methods confirmed the differences between the steady, activated and migratory states (Fig 3 a b c).

A notable feature of the steady state LCs is basal activity of several core signaling pathways that support homeostasis, maintain LCs within the healthy epidermal niche, and sustain low-level proliferation. These include the MAPK^32^, EGFR^33^, TGF-β^34^, WNT^35^, and P53^36^ pathways, as well as transcription factors of the Fos/Jun/AP-1 family^37^ (*Fos*, *Fosb*, *Junb*, *Jund*, *Fosl1*) (Fig 3 a c). Steady state LCs exhibit transcriptional signatures related to cell cycle progression, including pathways such as REGULATION_OF_CELL_CYCLE, G1/S_TRANSITION, G2/M_CHECKPOINT, and CHROMATIN_ORGANIZATION. The activated and migratory states LCs, demonstrate mTOR pathway^38^ changes, switching from mTORC2 to mTORC1, which is associated with changes in the expression of *Rictor*, *Riptor*, *Deptor*, and *Mtor* genes. The signaling landscape of migratory state LCs is defined by strong activation of cytoskeletal reorganization pathways, especially those involving actin dynamics controlled by the Rho family GTPases^39^ *Cdc42*, *Rac1*, and *RhoA* (Fig 3 b). In addition, during the transition from activation to migration, LCs express different sets of metalloproteases. *Mmp9*^40,41^ has elevated level in the steady state LCs while decreases in the activated and migratory states LCs, suggesting a role in basal matrix interaction. Upon activation, *Mmp14*, *Mmp20*, *Mmp19*, *and* Mmp23 also increase, supporting tissue remodeling and mobility. In the migratory state, *Mmp25* becomes upregulated, although it was not previously associated with LCs migration (Fig 3 b).

An additional characteristic observed was a change in energy balance as all three states had their own metabolic characteristics. In steady state LCs, the negative regulator of glucose metabolism *Txnip*^42^ is active. Also, transcripts of proteins responsible for metabolism and lipid transport^43^ are expressed (*Apoe*, *Abcg1*, *Abca1*, *Apba1*, and *Acsl5*) (Fig 3 b). Thus, steady state LCs predominantly rely on fatty acid oxidation^44^ (FAO) and moderate oxidative phosphorylation^45^ (OXPHOS). Activated state LCs switch to aerobic glycolysis, as indicated by enrichment of the glycolysis pathway and upregulation of key glycolytic enzymes, including *Eno1*, *Pfkfb3*, *Pfkfb4*, *Pkm*, and *Gapdh*^46,47^ (Fig 3 b). Mitochondrial metabolism also becomes engaged, with increased expression of *Gpd2*, *Gpx1*, *Ftl1*, and *Ehd1*, suggesting complementary oxidative support for energy and redox balance^48–50^ (Fig 3 b). At the same time, lipid metabolism remains active, and expands, highlighted by activation of *Fabp5*, *Apol7c*, *Cyp51*, and enrichment of the cholesterol homeostasis pathway^51,52^ (Fig 3 b). During the transition to the migratory state, glycolysis is progressively downregulated, as shown by decreased expression of key glycolytic enzymes^53^ such as *Hk2*, *Gpi1*, *Pfkp*, and *Ldha* (Fig 3 b). At the same time, the cells revert to OXPHOS as the primary source of energy, supported by the upregulation of mitochondrial genes including *Sdhaf1*, *Ndufa11*, *Cox5a*, and *Atp6v0e*.

Since LCs are antigen-presenting cells and, we paid special attention to the immunological pathways by which LCs perform their essential functions. The expression of multiple NF-κB components such as Rel and Nfkb1 gradually increases from the early activation state, peaking in the terminal migratory state^54^. STAT family members *Stat3*, *Stat4* and *Stat5*, which are classically associated with cytokine signaling, have the same pattern. The combination of pro-inflammatory and anti-inflammatory signals observed during LCs activation and migration suggests a finely regulated process. While components of the pro-inflammatory NF-κB pathway are upregulated^54^, the expression of *Nfkbia*, an inhibitor of NF-κB, is also increased^55^. Similarly, certain pro-inflammatory mediators such as *Il1b*^56^, *Il21r*, and *Cd137* are upregulated (Fig 3 b), while others including *Tnf*^57^, *Il1r1*, *Il13ra1*, *Il18*, and *Il1rap* are downregulated (Fig 3 b). At the same time, several anti-inflammatory genes, such as *Socs2*^58^, *Tnfrsf11b*, *Il4i1*, *Il10*, *Il1rn* show increased expression (Fig 3 b). This pattern likely reflects the need for precise control during LCs maturation and the extensive cellular remodeling that occurs during activation and migration. Regulating the inflammatory response may help prevent excessive activation while still supporting effective immune function. In addition, we observed increased expression of chemokines such as *Ccl22*, *Ccl17* and *Ccl5* (Fig 3 b), which are involved in recruiting T cells to lymphoid tissues^59,60^. These findings support the idea that LCs undergo a tightly balanced immune activation process that prepares them for antigen presentation while avoiding uncontrolled inflammation^61–63^.

Other interconnected but previously underexplored pathways that we examined in more detail include phagocytosis and the complement system, which emerge as key functional processes defining the activated state of LCs. Activated state LCs express bacterial and fungal pattern recognition receptors such as *Cd14* and *Tlr2, Tlr4, Tlr5*, *Clec7a, Clec4n, Clec4d, Nod2, Nlrc4, Nlrp3, Aim2* (Fig 3 b) along with key components involved in phagosome formation, including *Syk*^64^, *Vav1*^65^*, Hck*^66^ demonstrating overall increased PI3K signaling. Genes encoding the activator and core subunits of the proteasome^67^ and immunoproteasome^68^ such as *Psme*, *Psma*, *Psmc*, and *Psmd* (Fig 3 b) also begin to be expressed during activated state. In addition, *Fcgr2b* and *Fcgr3*, which facilitates the uptake of antibody-opsonized antigens^69^, is upregulated in activated state. LCs also express the “do not eat me” signal *Sirpa*^70^ at three all states. We observed a coordinated downregulation of genes related to MHC class II antigen presentation^71^ in activated and migratory states, including *Cd74*, *Ciita*, and *Marchf1* (Fig. 3 b). This downregulation likely reduces the turnover of MHC-II–antigen complexes on the LCs surface, facilitating effective antigen presentation to T-cells in lymph nodes^72^. In contrast, components of the MHC class I pathway and cross-presentation machinery remain active through activated and migratory states.

Another important feature of the activated state is the increased expression of genes associated with the complement system. Subunits of the C1q^73^ complex: *C1qa*, *C1qb*, and *C1qc* were upregulated (Fig. 3 b), suggesting engagement of the classical complement pathway. Unexpectedly, expression of properdin (*Cfp*), which stabilizes the alternative pathway^74^, was also detected (Fig. 3 b). LCs expressed neither the enzymes required for activation of the C1q complex nor the central components of the classical complement system, including C3^75^ and its converting enzymes. Despite the absence of full complement activation machinery, LCs expressed several complement receptors, including *Itgam* (*Cr3a*), *Itgax* (*Cd11c*), and *C3ar1* (Fig. 3 b), indicating a potential capacity to sense complement components. Expression of C5 (Hc) was observed at the late stage of migration, although neither its receptors nor the enzymes required for generating the anaphylatoxin C5a were detected (Fig. 3b).

Complement system components expression patterns suggest a possible role for the complement system in modulating LCs function, migration, or localization during activation.

#### TFs:

Transcription factor network analysis supports pathway and gene-level findings across LCs states. The steady state LCs is defined by factors involved in epidermal identity (*Tfap2e*^76^, *Ehf*^77^, *Yy1*^78^), moderate proliferation (*E2f1*^79^, *E2f7*, *Vdr*, *Junb*, *Jund*^80^), and baseline immune function (*Runx3*^81^, *Etv1*^82^, *Cebpa*^83^) (Fig. 3 a). The activated state is characterized by transcription factors *Spi1*, *Mafb*, *Cebpb*, and *Cebpe* known to drive myeloid activation and inflammation^84–87^, and by *Srebf2*, *Trps1*, *Bhlhe41*, and *Rfx4* which may support metabolic and transcriptional reprogramming^88–90^(Fig. 3 a). The migratory state is characterized by upregulation of *Irf1*^91^, *Irf7*^92^, *Irf8*^93^, *Stat3*, *Stat4*, and *Rel*, promoting antigen presentation^94–96^ and immune signaling (Fig. 3 a), along with *Runx2*, *Zeb1*, *Etv6*, and *Tead4*, which are linked to increased motility and epithelial-to-mesenchymal transition^97–99^.

### Separate clustering of the untreated sample revealed 6 homeostatic populations of LCs

We also independently analyzed and clustered the sample obtained from untreated mice to identify distinct homeostatic populations of Langerhans cells (LCs) (Extended Data Fig.1. c). In this untreated sample, we identified six populations, with the most common being fully quiescent LCs, followed by a prominent population characterized by a stress-responsive, immunomodulatory phenotype. Differential expression analysis (Supplementary Table 3) revealed that these immunomodulatory LCs express a range of heat shock proteins and chaperones (e.g., *Hsph1*, *Hspa1a*, *Hspa1b*, *Dnajb1*, *Dnajb4*, *St13*, *Bag3*, *Ubqln1*), along with immunoregulatory genes (*Il18r1*, *Il1rl2*, *Tnfaip8l2*, *Tnfaip8l3*, *Slamf7*, *Card9*, *Nfatc3*, *Sirpa*). These cells are distinct from the activated LC population, as they are located on the opposite side of the UMAP projection relative to the pre-activated cluster. Between the homeostatic and stress-responsive immunomodulatory populations, we identified a transitional cluster marked by the expression of *Mrnip*^100^, a known activator of DNA damage response signaling. The dividing LC population expressed a full spectrum of proliferation markers (*Mki67*, *Kif11*, *Cdk1*, *Mcm3*, *H1f4*, *Stmn1*). Finally, the migratory LCs in this sample displayed characteristics consistent with those described previously. Despite available differential expression data, we were unable to clearly determine the function of the population we termed stress-responded immunomodulatory LCs. Its role will be investigated in future studies.

### Cell–cell communication analysis identifies fibroblast–LCs signaling axis in cutaneous injury

**Fig. 4.**
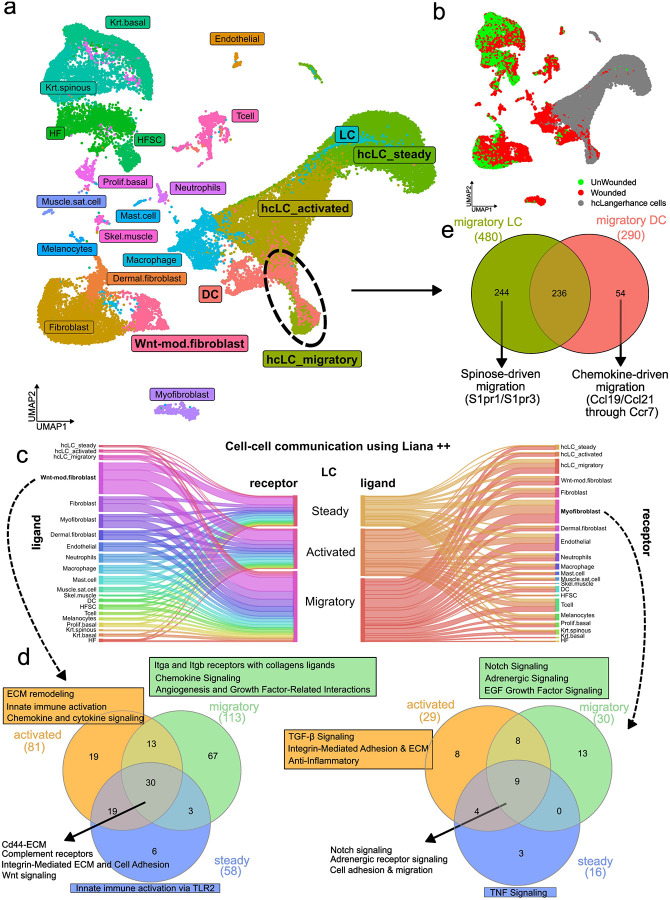
Interaction landscape of activated LCs in skin injury. a Integration of our dataset with Haensel et al. (2020) reveals transcriptional proximity between migratory DCs and migratory LCs; b UMAP visualization highlighting data from Haensel et al. (2022): wounded skin samples shown in red, unwounded in green. LCs data from this study are shown in grey; c Differential expression analysis between migratory DCs and LCs suggests distinct mechanisms underlying their respective migratory behaviors; d Cell–cell communication analysis using the LIANA++ tool indicates that LCs engage in the most extensive crosstalk with a population of wound-specific, WNT-modulated fibroblasts; e Interaction strength between LCs and WNT-modulated fibroblasts increases progressively during the transition from steady state to activation and migration.

To investigate how LCs interact with other skin resident cells, we integrated our data with the Haensel et al. (2020) dataset, which encompasses the majority of cell types under unwounded and wounded conditions. We then performed a cell-cell communication analysis with an integrated data set (Fig 4 a b). The dataset was integrated using Seurat, and ligand–receptor interactions were conducted with LIANA++ (Supplementary Table 2), that aggregates multiple ligand–receptor interaction databases. We found that LCs interact most extensively with dermal fibroblasts and myofibroblasts, particularly with a subset of WNT-modulated fibroblasts^101^ emerging in the context of tissue injury (Fig 4 c). Key interacting receptors on LCs include *Cd44*^102^, complement receptors, integrins, and WNT-related receptors, while fibroblasts predominantly express extracellular matrix components^103^(Fig 4 d). In the steady state, interactions with fibroblasts are dominated by homeostatic ECM components binding *Cd44*, like collagens, laminins and glycoproteins. Activation introduces integrin-mediated interactions between LCs and fibroblasts (e.g., *Itga9_Itgb1*) and immune-related signaling (innate immune, chemokine, cytokine), reflecting ECM remodeling and immune readiness. In the migratory state, LCs shift ECM interaction from *Cd44* to high-affinity^104^ integrins (*Itga9_Itgb1*, *Itga5_Itgb1*, *Itgav_Itgb1*). For myofibroblasts, the main interactions are mediated by Notch signaling and adrenergic signaling, which expand as LCs are activated and migrated (Fig 4 d).

Together, these progressive changes in LCs-fibroblasts interactions reflect a coordinated remodeling of the cell communication landscape, enabling the transition of LCs from tissue-resident maintenance to active immune engagement and migration.

### Divergent migratory mechanisms of LCs and dermal dendritic cells

In addition to cell–cell communication findings, we observed curious overlap between migratory state LCs and migratory dermal dendritic (DCs)^105^, converging into a single cluster (Fig 4 e). Both DCs and LCs are resident immune cells in the murine epidermis. Dermal DCs, a specialized subset of dendritic cells residing in the dermis, play a crucial role in initiating and regulating immune responses by capturing antigens and migrating to draining lymph nodes. Unlike epidermal LCs, dermal DCs are more heterogeneous and include subsets with distinct phenotypes and functions, such as pro-inflammatory and tolerogenic roles. These migratory populations shared a core gene expression program related to antigen presentation, immune activation, and lymph node homing, characterized by the expression of *Ccr7*, *Cd80*, *Cd86*, and *Irf8*. Despite this convergence, they retained distinct molecular features reflective of their origins and functions. Migratory DCs exhibit a strong interferon-stimulated gene signature, such as *Ifit1*, *Rsad2*, and *Oasl2*, indicating a heightened antiviral and pro-inflammatory profile. In contrast, migratory state LCs preserve epidermal and tissue-resident characteristics, marked by elevated expression of *Aldh1a2*, *Pparg*, and *Rora*, and produce regulatory cytokines such as *Il10* and *Il23a*, suggestive of an immune-modulatory role with potential Th17/Th2 skewing. Additionally, LCs express *S1pr1* and *S1pr3*, implicating sphingosine-1-phosphate signaling in their tissue egress^106,107^, in contrast to DCs, which predominantly utilize chemokine axis *Ccr7*–*Ccl19/21*^108^ (Fig 4 e). Collectively, these findings reveal a shared immunogenic core between LCs and DCs, while underscoring key differences in their migration strategies.

### Activated state LCs potentiate antigen-driven adaptive immune responses

**Fig. 5.**
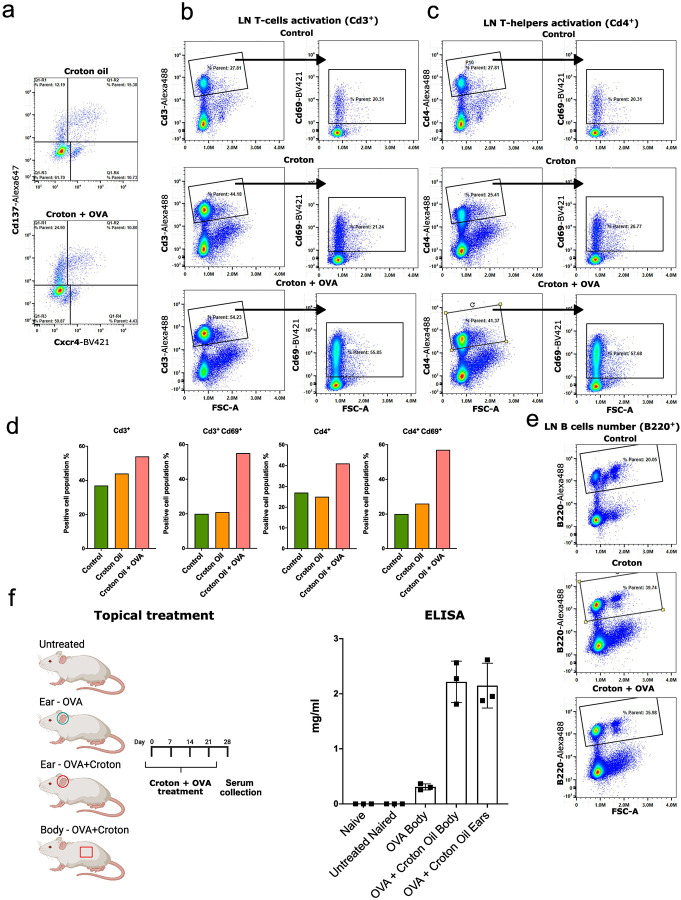
Simultaneous application of OVA and croton oil enhances immune activation; a The number of activated LCs increases significantly under combined treatment; b A higher number of activated CD3+CD69+ T cells is observed in the lymph nodes; c Phenotypic analysis confirms that the activated T cells are predominantly T helper cells; d Bar plot quantifying the total number of T cells across treatment conditions; e The number of B cells also increases in response to combined OVA and croton oil treatment; f Co-application of croton oil and OVA leads to robust production of anti-OVA IgG antibodies, in case of body treated 2 cm2, indicating a strong humoral immune response. All FACS experiments were performed in triplicate; data not shown.

As described before, activated state LCs express genes associated with phagocytosis and proteasome-mediated antigen degradation, key processes in immunological antigen presentation. To determine how the extent of LCs activation differs under antigen exposure, we compared the responses to croton/acetone treatment alone versus the same treatment with the addition of excess antigen, specifically ovalbumin (OVA)^109^. For this experiment, we established multiple groups of mice receiving different treatments (Fig 5 a). The groups were as follows: one mice received topical application of ovalbumin (OVA), the second group was treated with croton/acetone alone, and the third group received a combination of both OVA and croton/acetone. After 48 hours, flow cytometric analysis revealed a higher number of Cd137^+^ activated state LCs in the group treated with combination of both OVA and croton/acetone (Fig 5 b–d). This observation suggests improved OVA antigen uptake and processing capabilities in activated-state LCs following enhanced activation by croton/acetone^110^. To further investigate the immune response, we isolated lymph node cells from the all groups of mice. In the group that received both OVA and croton/acetone, there was a significant increase in Cd69^+^ T helper cells, indicating early T cell activation (Fig 5 b–d). Additionally, staining for B220 (*Cd45r*) revealed a higher presence of B cells in this sample, suggesting an augmented humoral immune response as well^111^ (Fig 5 e).

To evaluate the functional significance of observed phenomenon, we quantified an antibody production. We divided mice into four experimental groups and administered different combinations of OVA and croton/acetone treatments: untreated group, ears treated with OVA only, ears treated with both OVA and croton/acetone, 2 cm^2^ body treated with both OVA and croton/acetone (Fig 5 f). The treatment was carried out once every 7 days for one month, after a month the blood was collected from the mice and serum was isolated. The presence of IgG antibodies to OVA was analyzed using ELISA. We found that mice treated with both OVA and croton/acetone had a higher amount of anti-OVA IgG antibodies (Fig 5 g). This result findings highlight the functional relevance of the activated state of LCs in facilitating efficient antigen uptake and promoting systemic humoral immunity.

Altogether, these findings demonstrate that the activated state of LCs is not merely a transient intermediate, but a functionally distinct state with enhanced capacity for antigen sensing and immune priming. In addition, antigen exposure during this state significantly amplifies downstream adaptive immune responses, including both T cell activation and antibody production.

### Complement cascade to coordinate local immune responses by LCs attraction to wounded area

**Fig. 6.**
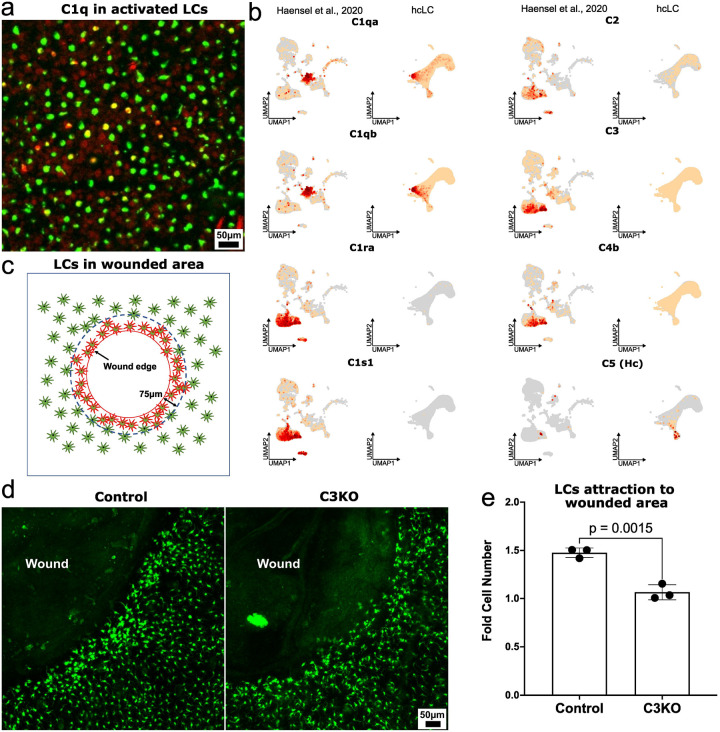
Role of complement signaling in LCs response to skin lnjury. a C1q expression in activated LCs 48 hours after croton/acetone treatment of ear skin; red – C1q, green – Lang-GFP; b Distinct epidermal cell types express different components of the complement system, indicating cell type–specific roles in complement activation and regulation; c Schematic representation of the 75 μm zone surrounding the wound; d Knockout of the central complement component C3 impairs the recruitment of activated LCs to the wound edge, highlighting the essential role of C3 in LC-mediated immune surveillance during tissue injury; e Comparison of LCs enrichment within a 75 μm circumferential zone around the wound in control and C3KO mice. Cell counts at 48 hours post-injury were compared with those immediately after wound creation in the same mouse. Bar plot statistics were calculated using a paired t-test (n = 3).

The transcriptomic analysis of LCs suggested the complement system as a key innate immune pathway involved in LCs regulation. In particular, activated state LCs express C1q subunits, which are central to initiating the classical complement pathway (Fig. 6 a)^112^. At the same time, fibroblasts, especially the WNT-modulated subset^113^, express *C1s1* and *C1ra*, serine proteases required for the activation of C1q (Fig. 6 b). This interaction supports the initiation of the complement cascade, promoting pathogen recognition and clearance through opsonization^114^. Furthermore, WNT-modulated fibroblasts express *C3*, a central component of the complement system, along with *C4b*, which is required for the conversion steps in the complement cascade^115^(Fig. 6 b). This expression pattern highlights the cooperative interaction between LCs and fibroblasts in coordinating a local immune response.

The *C3* derivative is an anaphylatoxin C3a, a known factor that attracts antigen presenting cells to the site of inflammation. It has never been described in the context of LCs despite the fact that the presence of an anaphylatoxin receptor in LCs is known^116^. To investigate the functional role of *C3* in complement activation and LCs recruitment, we generated *C3*-deficient (C3KO) mice combined with the *Lang-GFP* allele to visualize LCs with intravital imaging. We created 1 mm diameter circular wounds on the ears of both C3KO and control mice only express Lang-EGFP and used intravital microscopy to quantify LCs within a 75 μm region adjacent to the wound edge (Fig. 6 c). In C3KO mice, the number of LCs within 75 μm of the wound region remained unchanged 24 hours after injury, despite the fact that the LCs became activated and lost their regular pattern. In contrast, control mice only express Lang-EGFP without C3KO showed a 15–25% increase in LCs numbers within the same 75 μm zone 24 hours after injury (Fig. 6 d e), with the source of these cells likely being regions located more than 75 μm from the wound. As we showed before activated LCs reduce their ability to proliferate.

In conclusion, our findings establish the complement system—particularly *C3* and its downstream effectors—as critical regulators of LCs recruitment during skin injury. Importantly, the results show that the activated state of LCs is not only defined by transcriptional priming for antigen presentation but also reflects active crosstalk between LCs and the diverse cell populations present in the damaged or inflamed epidermis. However, not all of these interactions are detectable through transcriptome analysis alone, highlighting the need for high-quality data acquisition and integrative approaches to fully understand intercellular communication in inflamed skin. To support this, we present a comprehensive and high-quality transcriptomic dataset of LCs across all major epidermal states. Our findings expand the traditional view of LCs from passive antigen sentinels to dynamic regulators of immune coordination and tissue repair.

## Discussion

Epidermal LCs form a dense network responsible for sensing environmental pathogens and signals. LCs quantity and dynamic behavior suggest a significant functional role of these cells in skin immune surveillance. However, the transcriptomic dynamics and functional consequences of LCs activation remain incompletely understood. Applying scRNA-seq to murine epidermal LCs provides an unprecedented opportunity to explore LCs transcriptional diversity and functional specialization. In this study, we obtained a high-quality single-cell dataset of over 22,000 LCs encompassing the entire activation trajectory. These data capture the dynamic progression of LCs from the homeostatic steady state through an intermediate activated state to the fully activated migratory state. The presence of activated state has not been previously demonstrated.

Using intravital imaging, we identified a time window in which the epidermal LCs network was disrupted but cell numbers remained largely preserved. Traditional scRNASeq analysis combined RNA Velocity analysis of our dataset showed that LCs responded similarly to both the stamp-punch and croton/acetone treatments, demonstrating the robustness of our activation methods and the same reaction of LCs to physical and conditionally chemical stimuli, characterized by the preservation of the structure of the epidermis, but its impregnation with an agent that activates the immune response. This allowed us to isolate and characterize activated state LCs, a previously unrecognized intermediate population. We distinctly characterized the molecular features of activated state, separately from those of the migratory and steady states. The activated state LCs exhibited enhanced glycolytic activity, upregulation of complement system components, and increased expression of phagocytosis-related genes. These changes suggest a metabolic and functional shift that prepares LCs for both local immune catching and subsequent migration to lymphoid tissues. In addition, we identified that specific markers such as *Cd137*, *Ly6a*, and *Cd14* reliably distinguished the activated state LCs, providing an unprecedented basis for convenient activated LCs sorting in future studies.

Notably, we identified complement system components and their receptors as key markers of the activated and migratory states of LCs. We also observed diverse expression patterns of complement components across cell types: activated LCs express elements of the C1q complex, while fibroblasts produce the enzymes required for its activation. Wound-specific, WNT-modulated fibroblasts express the central component *C3* and enzymes necessary for its cleavage into the active anaphylatoxin C3a, whereas LCs express multiple receptors for *C3* cleavage products. Using C3-deficient mice, we demonstrated that C3 is essential for the effective recruitment of activated LCs to wound sites, suggesting that *C3* or its derivatives—originating locally from fibroblasts or systemically from circulation—guide LCs migration to tissue damage. This mechanism may help discriminate significant injury from minor insults, preventing unnecessary LCs activation.

In addition, we found that LCs transitioning to the migratory state begin expressing *C5*, the precursor of the potent anaphylatoxin C5a. This likely promotes recruitment of dermal immune cells to sites where LCs exit the epidermis, implying that LCs not only initiate adaptive immunity via lymph node migration but also coordinate local innate immune responses during early inflammation. Integration of our dataset with Haensel et al. (2020) revealed no alternative sources of *C5* in wounded skin, suggesting LCs are a primary source of C5 under pathological conditions. Clinical and in vivo evidence links *C5* cleavage products with inflammatory skin diseases such as psoriasis and atopic dermatitis^117–122^. Our findings propose that LC-driven complement activation contributes to immune cell recruitment and inflammation, offering new insights into skin immunobiology and potential therapeutic targets for inflammatory skin disorders.

Another interesting revealed aspect is the transcriptional similarity between migratory state LCs and migratory DCs. Integration of our data with the dataset from Haensel et al. (2020) showed that wound-specific activated DCs express *Cd14* and other phagocytosis-related markers. Notably, DCs phagocytic markers disappear at the stage when migratory DCs overlap with migratory state LCs. We found differences in the migration mechanisms of LCs and DCs. It seems that LCs migrate with an emphasis on the sphingosine-1-phosphate pathway, whereas dermal DCs predominantly use Ccr7 and its ligands. The differences between these cell populations and it’s migration mechanisms, as well as the biological significance of these observations, will be the subject of future investigations.

Based on our data, activated LCs by croton/acetone treatment were capable of phagocytosing antigen and inducing downstream adaptive immune responses, including antigen-specific antibody production. The functional relevance of this activation was confirmed by the induction of OVA-specific antibody production in croton/acetone-OVA mice. These results highlight the importance of delivering antigen to LCs, as well as precisely controlling their activation and the timing of functional transitions such as phagocytosis and migration.

In this study, we defined a distinct activation trajectory of epidermal LCs and identified a transcriptionally and functionally unique intermediate state characterized by enhanced phagocytic activity and complement engagement. These findings provide a foundation for understanding how activated LCs integrate immune sensing, antigen uptake, and migration during skin inflammation.

## Material and methods

### Mice

CD-1 mice were obtained from Charles River Laboratories. Lang-EGFP (JAX 016939) and C3 KO (JAX 029661) mice were obtained from were obtained from Jackson Laboratories. K14-H2BmCherry mice were obtained from V. Greco (Yale School of Medicine). To simultaneously visualize LCs, and epithelial cells, Lang-EGFP; K14H2B-mCherry mice were generated. To knock out C3 and visualize LCs, Lang-EGFP; K14H2B-mCherry; C3 KO^−/−^ mouse was generated. Siblings were used as controls (Lang-EGFP; K14H2B-mCherry; C3 KO^+/−^). Mice were housed in ventilated racks under controlled conditions, including an ambient temperature of 22 °C, relative humidity of 50% ± 10%, and a 12-hour light/dark cycle (lights on from 07:00 to 19:00). Both male and female mice from experimental and control groups were randomly selected for live imaging and LCs isolation. All animals used in this study were between 5 and 8 weeks old. Blinding was not employed. All animal procedures were conducted with approval from the Institutional Animal Care and Use Committee (IACUC) at Michigan State University and conducted in accordance with institutional and national guidelines for animal welfare.

### Invivo LCs activation (croton oil/acetone, stamp-punch)

Mice were anesthetized in an isoflurane chamber, and anesthesia was maintained throughout the experiment using vaporized isoflurane delivered via a nose cone, as previously described^123^. Fur was removed from the ears and head using Nair cream (Naircare; 022600003014) applied for 2 minutes using Q-tip, followed by tap water cleaning and a second 1-minute application. An electric razor was not used to avoid causing any physical damage to the skin. The croton oil solution was freshly prepared before each treatment by mixing croton oil (Sigma; C6719), acetone (Sigma; 179124), and olive oil (Sigma; O1514) in a 4:5:1 ratio. Mouse ears were carefully affixed and flattened onto a plastic cube (15 × 15 mm) using petroleum jelly (Vaseline). Additional petroleum jelly was applied at the base of the ears (proximal to the head) to prevent leakage of the solution. A total of 35 μL of the croton oil mixture was applied to the external surface of each ear and evenly distributed using a plastic pipette tip. Both ears were treated simultaneously. Following application, the mouse remained undisturbed for 10 minutes. The ears were then detached from the plastic cube, and the mouse was returned to the isoflurane chamber for an additional 10 minutes. This treatment procedure was repeated 24 hours later. Croton-oil activated state LCs were isolated 48 hours after the initial treatment. For physical treatment using microneedle puncture, a microneedle array consisting 38 needles, each 0.2 mm in length (Skinmedix; micro needle dermal stamp 0.2mm; 765573893595), was used. Fur removal and ear fixation were performed as described above. A total of 750 microneedle punctures (neat, without pressing) were applied to each ear, evenly distributed across the ears surface. The procedure was repeated 24 hours later with a reduced number of 150 punctures per ear. LCs from stamp-punched skin were isolated 48 hours after the first treatment.

### Epidermal single-cell suspension preparation and LCs sorting

Mice were treated as described above, while control mice received only Nair treatment at the same time as experimental animals. The procedure was performed 48 hours after the initial treatment in both the croton oil and microneedle punch. Mice were then euthanized following standard protocols after anesthesia with isoflurane. Subsequently, ears were excised, and the skin layer was carefully separated from the underlying cartilage. The isolated skin was placed epidermis-side up in a trypsin solution (0.3% trypsin (Thermofisher; 15090046) in 150 mM NaCl, 0.5 mM KCl and 0.5 mM glucose) and incubated for 1 hour and 45 minutes at 37 °C. Following incubation, the epidermis was gently separated from the dermis using fine forceps. The epidermal sheet was transferred to a scratched-wall plastic Petri dish (Fisher; FB0875711YZ) containing HBSS buffer supplemented with 10% FBS, trypsin inhibitor (Sigma; T6522) and DNaseI (Sigma; D4513), pre-warmed to 37 °C. The epidermis was then mechanically dissociated to single-cell suspension by pressing it against the scratched surface using forceps, producing cloudy cell suspension. This suspension was incubated for 15 minutes at 37 °C, then filtered through a 70 μm cell strainer. Dead cells were removed via magnetic separation (Miltenyibiotec; 130-090-101), followed by magnetic enrichment of LCs (Miltenyibiotec; 130-095-408). Cell viability was assessed using either trypan blue staining or flow cytometry (Supplementary protocol 1).

### Single cell library preparation and sequencing

For single-cell RNA sequencing, libraries were prepared according to the manufacturer’s manual using Chromium Next GEM Single Cell 3’ Kit v3.1 (10x Genomics, USA). And sequenced using 2 lanes (2.5GB) NovaSeq X 10B 100 SR (Illumina, USA).

### Single cells data analysis.

#### Preprocessing:

Raw scRNA-seq data were processed using Cell Ranger (v8.0, 10x Genomics) with default parameters and aligned to the GRCm39 reference genome, custom-modified to include additional coding sequences (CDS) for mCherry (AY678264.1) and eGFP (OQ870305.1).

#### Quality Control and Clustering:

Quality control, normalization, clustering, and visualization were performed using Seurat^124^ (v5.2) in R (4.4.0) and Scanpy^125^ (v1.10) with Python 3.10 for RNA Velocity and Cell-cell communication. In our quality control process, we filtered cells based on mitochondrial content and gene/UMI counts using the following Seurat parameters: nFeature_RNA > 1500 & nFeature_RNA < 7000 & nCount_RNA > 1500 & nCount_RNA < 40000 & percent.mt < 3.5. Data were normalized using NormalizeData, clustered using the Louvain algorithm, and visualized via UMAP. Given the LCs data’s homogeneity, UMAP was performed using the top 5 principal components. To avoid integration-related artifacts in our relatively homogeneous dataset and high quality, we initially analyzed LCs data without applying advanced integration algorithms. The resulting clustering pattern, including the division into three distinct clusters of LCs, was subsequently validated through integration with the dataset from Haensel et al., 2020.

#### Data Integration:

To integrate with Haensel et al., 2020, we used Seurat’s standard integration workflow. Anchors were identified across datasets using FindIntegrationAnchors, followed by IntegrateData to obtain a corrected expression matrix, which was used for downstream clustering and visualization.

### Pathway Analysis

SCPA^126^ (v1.6.1) was used to compute single-cell pathway activity scores using curated gene sets (MSigDB^127,128^, Reactome^129^, Wikipathways^130^)

#### Gene Regulatory Networks:

SCENIC^131^ (v0.12.1) was applied to infer transcription factor activity and regulons via GENIE3 (v1.28), RcisTarget (v1.26), and AUCell (v1.28).

#### RNA Velocity:

Velocyto^132^ (v0.17.15) and scVelo^133^ (v 0.2.5) was used for RNA velocity analysis, employing the dynamical model and projecting velocities onto UMAP.

#### Cell–cell Communication:

LIANA++^134^ (v0.1.12) was used to infer ligand–receptor interactions across cell types using consensus rankings from multiple methods.

### Intravital imaging

Mice were anesthetized using an isoflurane induction chamber, and anesthesia was maintained throughout imaging via a nose cone delivering vaporized isoflurane, as previously described33. Imaging was performed using Leica TCS SP8 X Two-Photon microscope equipped with Spectra physics Insight MP Laser and controlled via Leica LAS X software. Z-stack images were acquired using a ×25 water-immersion objective (NA 1.0; Leica), with 0.59 × 0.59 mm^2^ field of view. Excitation wavelengths were adjusted depending on fluorophore combinations: 940 nm for Lang-EGFP. Z-stacks were acquired in 2 μm steps to cover depths up to 80 μm. For large-area imaging, 9–12 tiled optical sections were acquired using a motorized stage with custom Fiji^135^ (v 1.53) stitching script.

### Voronoi diagram and Clark-Evance index

Spatial distribution of cells was analyzed using Voronoi tessellation in Fiji with the Voronoi plugin. Binary masks of cell centroids were generated, and Voronoi diagrams were computed. The area of each Voronoi cell was measured and used to assess spatial dispersion and local cell density. To quantify the degree of spatial regularity or clustering, the Clark–Evans index (R) was calculated using the Spatstat^136^ package (v 3.3) in R. Cell centroid coordinates were extracted and modeled as a point pattern. The index compares the observed mean nearest-neighbor distance to the expected value under complete spatial randomness (CSR). Values of R > 1 indicate regularity, R < 1 suggest clustering, and R ≈ 1 implies randomness.

### Flow cytometry

For flow cytometric analysis of LCs markers, the staining protocol was performed prior to the 70 μm filtration step. Draining mandibular lymph node (LNs) were isolated as previously described^137^. LNs cells suspension were prepared using the same procedure as for LCs, excluding the trypsin digestion step. LNs were mechanically dissociated by pressing it against the scratched surface using forceps, producing cloudy cell suspension. Following preparation of the cell suspension (from epidermis or LNs), the desired number of cells was aliquoted into a 96-well polypropylene v-bottom plate (Corning; 3344). Dead cells were stained using Live/Dead Blue dye (Thermofisher; L34961; 1:800) for 15 minutes at room temperature, followed by two washes with staining buffer. To block nonspecific binding, Fc receptors were blocked using Fc block reagent (BDbiosciences; 553142) for 15 minutes. Next, cells were incubated with nonconjugated, fluorochrome or biotin-conjugated primary antibodies (1:200 dilution) for 30 minutes in the dark (Biolegend: 108103, 123305, 104703, 146511, 100212, 100425, 104545, 103228). After two additional washes with staining buffer, cells were incubated with either (1:800 dilution) streptavidin-conjugated fluorochrome or appropriate fluorochrome-conjugated secondary antibodies (Biolegend: 405237). Following staining, cells were washed several times with staining buffer. Cells were then fixed with 4% paraformaldehyde in PBS for 15 minutes, washed three times with flow cytometry buffer, and subsequently analyzed by flow cytometry. Flow cytometry was performed using the Cytek Aurora spectral flow cytometer. Cells were acquired using SpectroFlo software. Compensation and unmixing were conducted automatically based on single-stain controls using auto fluorescence exclusion function. Data were exported in FCS 3.1 format. Flow cytometry data were analyzed using FCS Express (v 7.24, De Novo Software). Initial gating was performed to exclude debris, doublets, and dead cells. Subsequent gating strategies were applied based on marker expression to identify cell populations of interest. Data visualization included biaxial plots and population statistics.

### Ovalbumin treatment

To deliver ovalbumin (OVA) to LCs, OVA (Invivogen; vac-stova) was diluted in PBS to a concentration of 10 mg/m. Mice were anesthetized and immobilized; fur was removed from the ear and head, which was then flattened and secured onto a plastic support cube, as described above. A total of 70 μL of 100% absolute ethanol (Sigma; 459836) was applied to each ear surface. Immediately afterward, 30 μL of the OVA-PBS solution was applied and evenly distributed across each ear. The ear with ethanol – OVA mix was then dried for 10 minutes using a stream of argon gas to fix the protein onto the skin surface. After drying, a stable film of ovalbumin formed on the skin surface. Following drying, a mixture of croton oil, acetone, and olive oil (prepared as described above) was applied to the ear and left in place for 10 minutes. Mice were then transferred to isoflurane chamber an additional 10 minutes. For topical treatment of the body, a 10 × 20 mm bath was formed on the lateral side of the mouse using adhesive tape. The edges of the tape were sealed with petroleum jelly to prevent leakage and ensure localized application. OVA and a mixture of croton oil were applied in the same way as for the ear.

### Whole mount staining

Permeabilized whole-mount staining of full-thickness skin was performed in 24-well plates, following previously described protocol^138^ using anti-C1q antibody (Abcam: ab182451) and Alexa633 secondary (Invitrogen: A-21070).

### ELISA

Mouse Serum was isolated using the gel centrifugation method and then frozen for simultaneous analysis using by standard mouse Anti-OVA IgG enzyme-linked immunosorbent assay (ELISA) following the manufacturer’s protocol (Chondrex; 3011).

### Skin Wound

To create a round wound, we used a punch biopsy tool with a 1 mm diameter circular blade. Mice were anesthetized with isoflurane as previously described. A circular wound was made with a single light motion, carefully avoiding penetration into the dermis. The epidermal layer within the wound area was then gently removed using tweezers.

## Supplementary Material

Supplement 1 Extended data Fig. 1. From data filtering to functional insights. a Quantitative and qualitative assessment of data before and after filtering; b UMAP clustering results before and after filtering; c Separate clustering of the untreated sample reveals multiple homeostatic populations of LCs; d Summary of potential interactions between the complement system and LCs. LCs express specific components such as C1q and C5, while fibroblasts—particularly wound-specific subsets—express C3 along with the enzymes required for the activation and conversion of C1q, C3, and C5.

Supplement 2

Supplement 3

Supplement 4

Supplement 5

## Data Availability

All sequencing data generated in this study have been deposited in the Gene Expression Omnibus (GEO) under accession number GSE296148. Additional datasets: filtered and unfiltered data objects, integration and corresponding 10x Loupe browser files are available on Zenodo at https://doi.org/10.5281/zenodo.15319340.
