# An intermediate activation state primes Langerhans cell migration from the epidermis

**DOI:** 10.1101/2025.05.29.656912

**Published:** 2025-09-17

**Authors:** Artem Kiselev, Axel D. Schmitter-Sánchez, Sharod Williams, Sangbum Park

**Affiliations:** 1 Institute for Quantitative Health Science and Engineering (IQ), Michigan State University, East Lansing, MI, USA.; 2 Cell and Molecular Biology Program, College of Natural Science, Michigan State University, East Lansing, MI, USA; 3 RTSF Genomics Core, Michigan State University, East Lansing, MI, USA.; 4 Division of Dermatology, Department of Medicine, College of Human Medicine, Michigan State University, East Lansing, MI, USA.; 5 Department of Pharmacology and Toxicology, College of Human Medicine, Michigan State University, East Lansing, MI, USA.

**Keywords:** Langerhans cells, Single-Cell RNA Sequencing, Activation, Wound, Complement cascade

## Abstract

Langerhans cells (LCs) are a specialized subset of dendritic cells in the epidermis, forming a dense network that acts as a frontline defense through immune surveillance. Upon antigen uptake, LCs become activated and orchestrate subsequent immune responses by migrating to lymphatics. However, how transcriptional programs are regulated during activation and how LCs behave in vivo during this transition remain poorly understood. Here, we combine single-cell transcriptomic analysis and intravital imaging to reconstruct the activation trajectory of epidermal LCs. We present a high-resolution single-cell transcriptomic dataset of over 22,000 high-quality epidermal LCs in both homeostatic and injured conditions. We define specific LC subpopulations representing sequential activation stages, characterized at the level of pathways and transcription factors. Notably, we identify a distinct intermediate state that precedes their migration. Integrating our data with an external dataset from homeostatic and injured skin reveals that wound-specific, WNT-modulated fibroblasts are the primary source of C3, the central component of the complement cascade. Intravital imaging of C3-deficient mice demonstrated that C3 is essential for effective recruitment of activated LCs to wound sites. Together, our findings uncover a novel population of activated epidermal LCs and highlight complement signaling as a critical mediator of LC recruitment during skin injury.

## Introduction

Langerhans cells (LCs) are a specialized subset of dendritic cells that form a dense and regular organization in the epidermis^1^. They function as immune sentinels by detecting environmental antigens, sensing tissue damage, and initiating immune responses and antigen delivery to lymph nodes^2^. LCs have been extensively studied in their development, differentiation, and immunological roles, including antigen presentation. However, it remains unclear how LCs are activated in vivo and what functional consequences this has.^3-5^.

Recent studies have enhanced our understanding of LC biology, revealing previously unrecognized heterogeneity and functional specialization within this cell type^6,7^. For instance, Kim et al. (2024) identified four distinct LCs subpopulations in human skin: two steady-state and two pro-inflammatory/mature subsets. Notably, pro-inflammatory subsets were enriched in atopic dermatitis lesions, suggesting a role in disease pathogenesis^8^. Further, spatial and single-cell transcriptomic analyses have delineated communication networks between LCs and other skin-resident cells, such as keratinocytes and fibroblasts. These studies have uncovered how LCs interact with their microenvironment to maintain skin homeostasis and respond to inflammatory stimuli^9^. In murine models, single-cell RNA sequencing (scRNA-seq) has revealed that LCs exhibit a dual identity, functioning both as tissue-resident macrophages and as migrating antigen-presenting cells. This duality is influenced by intrinsic factors and the epidermal niche, highlighting the complexity of LC ontogeny and function^9^. Indeed, scRNA-seq has enhanced our ability to dissect LCs cellular landscapes, enabling high-resolution profiling of individual LC populations within the epidermis^10^. On the other hand, intravital imaging, combined with genetic modification and fluorescent protein labeling, offers powerful tools for real-time monitoring of cells in live animals. This is particularly valuable for studying LCs, one of the few immune cells whose behavior can be observed in an intact organism and directly compared under different conditions and treatments by imaging analysis.

Here, we combined high-resolution single-cell RNA sequencing with intravital imaging to reconstruct the activation trajectory of epidermal LCs under homeostatic and injury conditions in vivo. We identified distinct LCs subsets with unique gene expression signatures, uncovering functional diversification within the injured epidermis. Notably, we characterized discrete LC populations in both homeostatic and injured skin. Our work provides insights into their dynamic behaviors and functional roles in orchestrating the immune barrier response to tissue injury.

## Results

### Physical damage and chemical treatment causes similar LCs activation trajectory

In non-inflamed skin, LCs form a tight network across the epidermis, with cells maintaining relatively equal spacing^11,12^. This organization supports efficient immune surveillance and is sustained through local proliferation of resident LCs under homeostatic conditions. However, in skin insults, LCs lose their regular spatial distribution. This disorganization likely facilitates migration to the draining lymph node to initiate adaptive immune responses^13^.

**Fig. 1.**
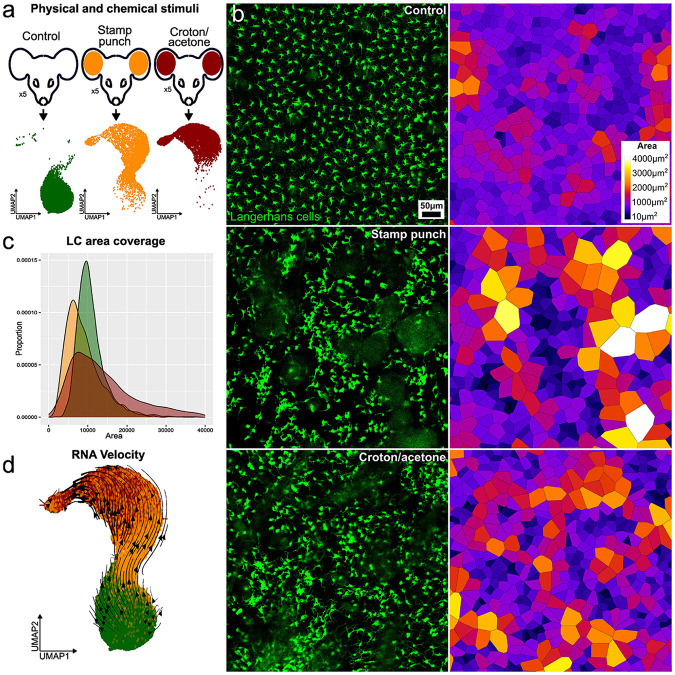
Experimental design and *in vivo* dynamics of LC activation and migration. **a.** Schematic overview of the experimental design for single-cell RNA sequencing (scRNA-seq) data acquisition (each group pooled from 5 mice). **b.** Representative images of intravital imaging of mouse ear epidermis under three conditions: untreated control, 48 hours post-croton oil treatment, and 48 hours post-stamp-punch stimulation. Corresponding Voronoi diagrams illustrate changes in LC organization (n = 3 mice). **c.** Distribution of cell territories derived from Voronoi diagram analysis highlights significant spatial differences between control and treated samples (n = 3 mice). **d.** RNA velocity analysis reveals the directional trajectory of LCs, supporting the sequential progression from steady state to activated and migratory states.

To define the changes in LC distributions as they become activated and migrate out of the epidermis from a homeostatic state in vivo, we used intravital microscopy with transgenic mice expressing GFP under the control of the Cd207 (Langerin) promoter^14^. We tested whether different types of stimuli lead to distinct perturbations in LC distribution. The first method, classified as physical, involved repeated puncturing of ear skin using a 0.2 mm microneedle array, referred to as the stamp-punch. The second method, classified as chemical, involved a 10-minute treatment of ear skin with a mixture of croton oil^15^, acetone, and olive oil, referred to as croton/acetone (Fig. 1a, b). Because fully activated LCs leave the epidermis, we sought to define the time when their spatial distribution becomes perturbed, but while they remain within the epidermis. Interestingly, in both physical damage and chemical treatment, LC activation peaked around 46 hours post-treatment, with disrupted spatial patterns but a cell number comparable to the homeostatic state (Fig. 1b, c).

Next, we examined transcriptomic changes caused by physical damage and chemical treatment with scRNA-seq using the 10x Genomics platform. We isolated LCs from three experimental groups: two corresponding to the activation methods described above and one untreated control group. Each scRNA-seq sample consisted of pooled epidermal LCs extracted from the ear skin of five mice. Single-cell analysis reveals a continuous trajectory reflecting progressive activation and migration of LCs; this trajectory is evident when examining the three groups (treated and control) together (Fig. 1a, c). To validate this finding, we applied RNA velocity^16^ analysis. These approach show that, in response to both physical damage and chemical treatment, LCs first transition into a distinct activated state before progressing to a migratory state (Fig. 1d). The trajectory appears to be unidirectional and irreversible over the 46-hour observation period, regardless of whether the stimulus is physical or chemical, indicating a single, shared activation program. We observe differences only in the extent of activation, reflected by varying enrichment in specific experimental groups.

Together, our intravital imaging and transcriptomic analyses demonstrate that both physical and chemical stimuli induce a common activation trajectory in LCs. Despite distinct modes of tissue insult, LCs exhibit synchronized spatial disorganization at 46 hours and progress through a shared, unidirectional activation-to-migration pathway without evidence of divergent transcriptional programs. These findings suggest that epidermal LCs utilize a conserved activation mechanism in response to diverse environmental challenges.

### Identification of an intermediate state of LCs preceding epidermal exit

**Fig. 2.**
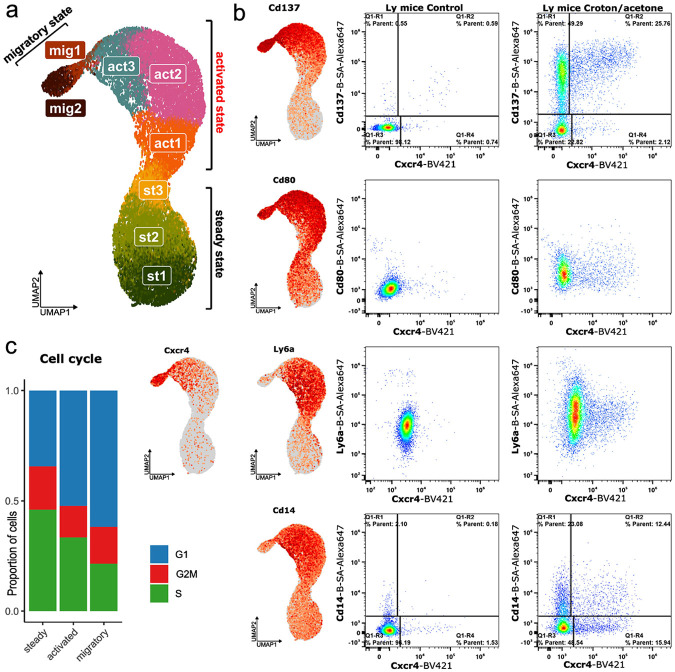
Transcriptional and functional characterization of LCs activation state. **a.** scRNA-seq analysis identified three major transcriptional states of LCs —steady, activated, and migratory — separated by UMAP clustering. For in-depth regulon and pathway analysis, the dataset was further subdivided into eight transcriptionally distinct substages. **b.** Differential gene expression and flow cytometry analysis demonstrates that activated LCs can be distinguished from steady and migratory populations using surface markers such as Cd137, Cd14, and Ly6a. Flow cytometry is representative data (n = 3 mice). **c.** During activation, the majority of LCs exit the proliferative cycle and undergo mitotic arrest, indicating a functional shift from self-renewal to immune engagement.

We next sought to clearly define the LC subpopulations during responses to stimuli and found three distinct clusters indicated by UMAP (Fig. 2a). Steady state LCs, representing quiescent cells in healthy/untreated epidermis, exhibit high-level expression of *Cd207*^17^, as well as low levels of *Cd80*^18^ and *Cd86*^19^ markers, associated with activation of antigen presenting cells. The migratory state, identified by high expression of *Cxcr4*^20^ and *Ccr7*^21^, forms the smallest cluster of cells that have almost left the epidermis, representing approximately 5% of the dataset (Fig. 2b). Between the steady-state and migratory populations we identified an intermediate activated LC population, a transitional LC state that has not been well identified^7,22,23^.

Next, we aimed to further characterize the LC populations by interrogating specific markers for each state, with a particular focus on the activated population. We combined bioinformatic analysis with flow cytometry-based protein-level validation, and using differential expression analysis we identified unique markers of each LC population as well as shared features among groups. Our analysis identified approximately 400 transcripts that clearly separate the activated state LCs from the steady and/or migratory states (Supplementary Table 1). Among these transcripts, we noted upregulation of well-established antigen-presenting cell activation markers, such as *Cd80* and *Cd86*, during both the activated and migratory states (Fig. 2b). In addition, we find that during the transition to the activation and migration states, LC proliferation activity decreases and most LCs enter the G1 stage^24^ (Fig. 2c). However, analysis of both transcriptomic data and flow cytometry results showed that these markers do not clearly discriminate activated and migratory states from steady state. To address this limitation, we prioritized identification of alternative markers that more precisely delineate the transitions between steady, activated, and migratory states at both the transcriptomic and protein levels. From transcripts specific to the activation state, we shortlisted 12 candidate genes based on predicted cell surface localization or roles as transcription factors. These transcripts include *Cd14*^25^, *Ly6a*^26^, *Il1b, Cd137* (*Tnfrsf9*)^27^, Ccr*7* (*Cd197), Cd49d* (*Itga4*), *Cd274*^28,29^, *Cxcr4, Adgre4* (*Emr4*), *Mafb*^30^, and *Bhlhe40*^31^. We focused specifically on transcripts with low or undetectable expression in one or more states, prioritizing those that either showed a progressive increase from the steady to migratory state or were selectively expressed in a single state with minimal expression in others. To validate these findings at the protein level, we performed flow cytometry using specific antibodies (Fig. 2b). We achieved the most pronounced separation of LC populations using a combination of surface markers, including the new LC activation markers (*Cd137, Ly6a*, and *Cd14*) that we describe above, along with the known migratory marker *Cxcr4* (Fig. 2b). We found that the combination of biotinylated *Cd137* and fluorochrome-conjugated *Cxcr4* provides the clearest discrimination of the activated state LC population (Fig. 2b). In this setup, *Cd137*-negative cells correspond to steady state LCs, while *Cd137*^+^ cells represent activated state LCs transitioning toward the migratory state. Additional markers, *Cd14* and *Ly6a*, also exhibited state specific expression patterns, peaking in the activated state and subsequently declining as cells advanced toward migration (Fig. 2b).

Collectively, our findings reveal a previously unknown intermediate activation state of LCs that bridges the transition from steady state to migratory state. Integrated transcriptomic and protein analyses identified specific markers that distinguish this transient population. Our characterization provides a framework for precise identification of this state and a deeper understanding of the full LC activation trajectory.

### Transcriptional and functional reprogramming of LCs activation

**Fig. 3.**
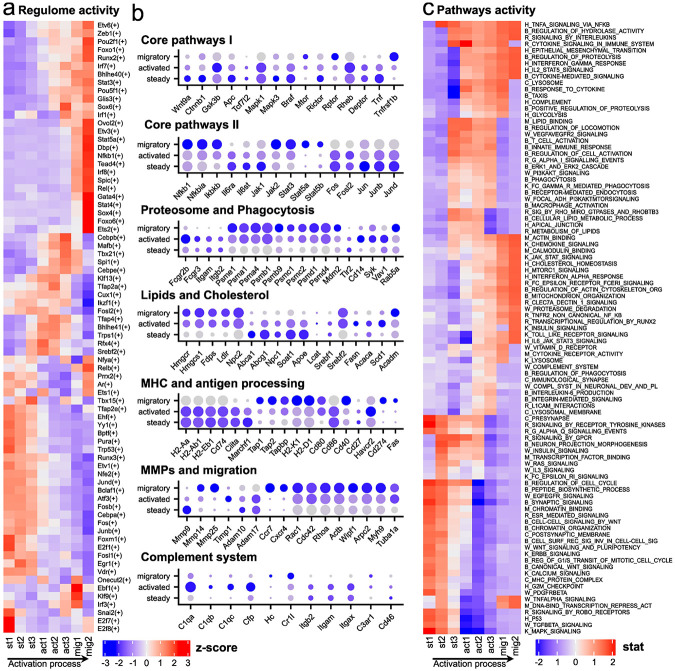
Regulatory network and pathway dynamics underlying LC activation and migration. **a.** Heatmap generated using the SCENIC tool, where red indicates high activity and blue indicates low activity by z-score of gene regulatory networks associated with specific transcription factors. **b.** Dot plots showing the relative expression of key genes during the activation and migration of LCs. Dot size reflects the proportion of cells expressing the gene, while color intensity represents expression levels. **c.** Pathway enrichment analysis using the SCPA package. Red indicates high pathway activity, and blue indicates low activity. Prefixes before pathway names denote the source database: H – Hallmarks, B – GO: Biological Processes, M – GO: Molecular Functions, C – GO: Cellular Components, R – Reactome, W – WikiPathways, and K – KEGG.

To uncover the key regulatory mechanisms driving transitions between LC states precisely, we subdivided the three states into eight transcriptionally distinct substates. We then applied transcription factor network activity analysis using SCENIC and assessed regulatory signaling dynamics through single-cell pathway analysis (SCPA), enabling us to track coordinated pathway changes across the LC activation continuum. Both of these approaches clarified the differences between the steady, activated, and migratory states (Fig. 3a-c).

A notable feature of steady state LCs is basal activity of several core signaling pathways that support homeostasis, maintain LCs within the intact epidermal niche, and sustain proliferation. These include the MAPK^32^, EGFR^33^, TGF-β^34^, WNT^35^, and P53^36^ pathways, as well as transcription factors of the Fos/Jun/AP-1 family^37^ (*Fos, Fosb, Junb, Jund, Fosl1*). Steady state LCs exhibit transcriptional signatures related to cell cycle progression from the pathway enrichment analysis (Fig. 3a, c). Activated and migratory state LCs exhibit mTOR pathway^38^ changes, switching from mTORC2 to mTORC1. The signaling landscape of migratory state LCs is defined by strong activation of cytoskeletal reorganization pathways, especially those involving actin dynamics controlled by the Rho family GTPases^39^, including *Cdc42, Rac1*, and *RhoA* (Fig. 3b). In addition, during the transition from activation to migration, migratory LCs highly express several metalloproteases, including *Mmp14* and *Mmp25*, suggesting a role in promoting LC migration through the tissue and into the lymphatic system. (Fig. 3b). These findings highlight dynamic signaling reprogramming that enables LCs to transition from tissue residency to migration.

Interestingly, we observe state-specific changes in metabolic characteristics. In steady state LCs, the negative regulator of glucose metabolism *Txnip*^40^ is active. Also, transcripts of proteins responsible for metabolism and lipid transport^41^ are expressed, such as *Apoe, Abcg1*, and *Abca1* (Fig. 3b). Thus, steady state LCs predominantly rely on fatty acid oxidation^42^ (FAO) and moderate oxidative phosphorylation^43^ (OXPHOS). Activated state LCs switch to aerobic glycolysis, as indicated by enrichment of the glycolysis pathway and upregulation of key glycolytic enzymes, including *Eno1, Pfkfb3, Pfkfb4, Pkm*, and *Gapdh*^44,45^ (Fig. 3b). Mitochondrial metabolism also becomes engaged, with increased expression of *Gpd2, Gpx1, Ftl1*, and *Ehd1*, suggesting complementary oxidative support for energy and redox balance ^46-48^ (Fig. 3b). At the same time, lipid metabolism remains active, and expands, highlighted by activation of *Fabp5, Apol7c, Cyp51*, and enrichment of the cholesterol homeostasis pathway ^49,50^ (Fig. 3b). During the transition to the migratory state, glycolysis is progressively downregulated, as shown by decreased expression of key glycolytic enzymes^51^ such as *Hk2, Gpi1, Pfkp*, and *Ldha* (Fig. 3b). At the same time, the cells revert to OXPHOS as the primary energy source, supported by upregulation of mitochondrial genes including *Sdhaf1, Ndufa11, Cox5a*, and *Atp6v0e*. Our data demonstrate that LCs undergo dynamic metabolic shift to meet the distinct energetic demands during the activation process.

Recognizing LCs as key immune cells, we investigated the regulation of immunological pathways during their state transitions. Expression of multiple NF-κB components gradually increases from the early activation state, peaking in the terminal migratory state^52^. We found that pro-inflammatory and anti-inflammatory signals coexist during LC activation and migration. While components of the pro-inflammatory NF-κB pathway are upregulated^52^, the expression of *Nfkbia*, an inhibitor of NF-κB, is also increased^53^. Similarly, certain pro-inflammatory mediators such as *Il1b*^54^, *Il21r*, and *Cd137* are upregulated (Fig. 3b), while others including *Tnf*^55^, *Il1r1, Il13ra1, Il18*, and *Il1rap* are downregulated (Fig. 3b). At the same time, several anti-inflammatory genes, such as *Socs2*^56^, *Tnfrsf11b, Il4i1, Il10, Il1rn* show increased expression (Fig. 3b). This pattern likely reflects the need for precise control during LC maturation and the extensive cellular remodeling that occurs during activation and migration. Regulating the inflammatory response may help prevent excessive activation while still supporting effective immune function. In addition, we observe increased expression of chemokines such as *Ccl22, Ccl17* and *Ccl5* (Fig. 3b), which are involved in recruiting T cells to lymphoid tissues^57,58^. These results support the idea that LCs undergo a tightly balanced immune activation process that prepares them for antigen presentation while avoiding uncontrolled inflammation^59-61^.

Given that LCs are antigen-presenting cells, we examined the phagocytic activity of LCs across different states. Activated state LCs express several bacterial and fungal pattern recognition receptors along with key components involved in phagosome formation, including *Syk*^62^ and *Vav1*^63^. Genes encoding the activator and core subunits of the proteasome^64^ and immunoproteasome^65^, such as *Psme, Psma, Psmc*, and *Psmd*, also begin to be expressed during activated state (Fig. 3b). In addition, *Fcgr2b* and *Fcgr3*, which facilitates the uptake of antibody-opsonized antigens^66^, is upregulated in the activated state. We observe a coordinated downregulation of genes related to MHC class II antigen presentation^67^ in activated and migratory states, including *Cd74, Ciita*, and *Marchf1* (Fig. 3b). This downregulation likely reduces turnover of MHC-II–antigen complexes on the LCs surface, facilitating effective antigen presentation to T cells in lymph nodes^68^. In contrast, components of the MHC class I pathway and cross-presentation machinery remain active through activated and migratory states. Our study provides a detailed molecular view of LC phagocytic function, highlighting how antigen uptake and processing are regulated across distinct activation states.

We also found that genes associated with the complement system are differentially expressed during LC activation. Subunits of the C1q^69^ complex, *C1qa, C1qb*, and *C1qc*, are upregulated (Fig. 3b), suggesting engagement of the classical complement pathway. Unexpectedly, we also detect expression of properdin (*Cfp*), which stabilizes the alternative pathway^70^ (Fig. 3b). LCs express neither the enzymes required for activation of the C1q complex nor the central components of the classical complement system, including C3^71^ and its converting enzymes. Despite the absence of full complement activation machinery, LCs express several complement receptors, including *Itgam* (*Cr3a*), *Itgax* (*Cd11c*), and *C3ar1* (Fig. 3b), indicating a potential capacity to sense complement components. We observed expression of C5 (Hc) at the late stage of migration, although neither its receptors nor the enzymes required for generating the anaphylatoxin C5a were detected (Fig. 3b). These intriguing expression patterns in complement system components suggest a role for the complement system in modulating LCs function, migration, or localization during activation. Our results suggest that LCs, in coordination with other cell types, modulate the complement system as part of their activation process.

### Separate clustering of the untreated sample reveals six homeostatic populations of LCs

We also independently analyzed and clustered cells from untreated mice to identify distinct homeostatic populations of LCs (Supplementary Fig.1). In this untreated sample, we identified six populations, with the most common being fully quiescent LCs, followed by a prominent population characterized by a stress-responsive, immunomodulatory phenotype. Differential expression analysis (Supplementary Table 3) revealed that these immunomodulatory LCs express a range of heat shock proteins and chaperones (e.g., *Hsph1, Hspa1a, Hspa1b, Dnajb1, Dnajb4, St13, Bag3, Ubqln1*), along with immunoregulatory genes (*Il18r1, Il1rl2, Tnfaip8l2, Tnfaip8l3, Slamf7, Card9, Nfatc3, Sirpa*). These cells are distinct from the activated LC population, as they are located on the opposite side of the UMAP projection relative to the pre-activated cluster. Between the homeostatic and stress-responsive immunomodulatory populations, we identified a transitional cluster marked by the expression of *Mrnip*^72^, a known activator of DNA damage response signaling. The dividing LC population expressed a full spectrum of proliferation markers (*Mki67, Kif11, Cdk1, Mcm3, H1f4, Stmn1*). In short, our data demonstrated that the heterogeneity reflects intrinsic diversity within homeostatic LCs, but is distinct from the activation-associated states.

### Cell–cell communication analysis identifies a fibroblast–LCs signaling axis in cutaneous injury

**Fig. 4.**
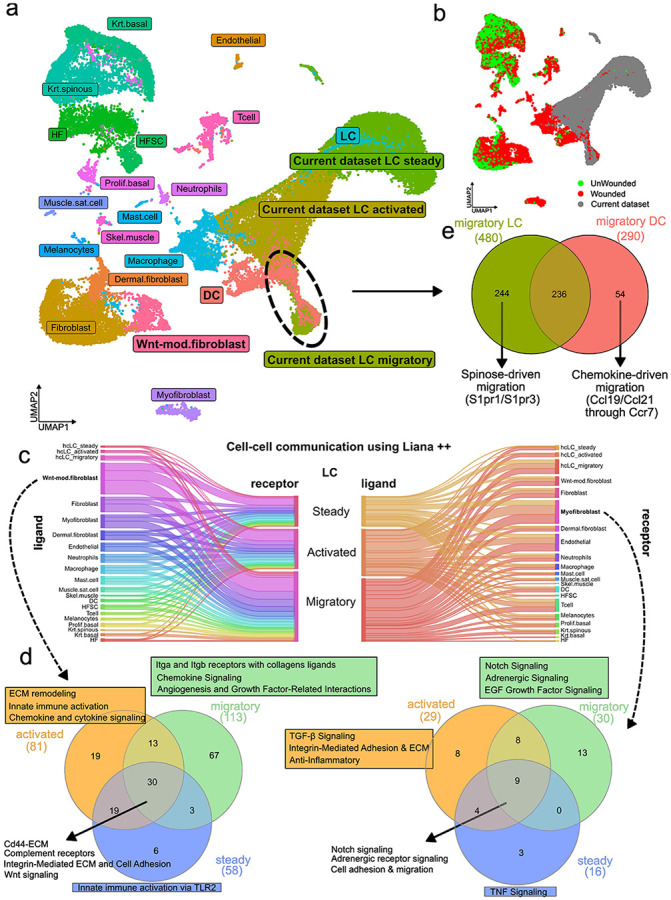
Interaction landscape of activated LCs in skin injury. **a.** Integration of our dataset with Haensel et al. (2020) reveals transcriptional proximity between migratory DCs and migratory LCs. **b.** UMAP visualization highlighting data from Haensel et al. (2022): wounded skin samples shown in red, unwounded in green. LC data from this study are shown in grey. **c.** Cell–cell communication analysis using the LIANA++ tool indicates that LCs engage in the most extensive crosstalk with a population of wound-specific, WNT-modulated fibroblasts. **d.** Interaction strength between LCs and WNT-modulated fibroblasts increases progressively during the transition from steady state to activation and migration. **e.** Differential expression analysis between migratory DCs and LCs suggests distinct mechanisms underlying their respective migratory behaviors.

To investigate how LCs interact with other skin resident cells, we integrated our data with the Haensel et al. (2020) dataset, which encompasses the majority of cell types under unwounded and wounded conditions. We then performed a cell-cell communication analysis with these integrated data (Fig. 4a, b). The dataset was integrated using Seurat, and ligand–receptor interactions were identified with LIANA++ (Supplementary Table 2), which aggregates multiple ligand–receptor interaction databases. We find that LCs interact most extensively with dermal fibroblasts and myofibroblasts, particularly with a subset of WNT-modulated fibroblasts^73^ emerging in the context of tissue injury (Fig. 4c). Key interacting receptors on LCs include *Cd44*^74^, complement receptors, integrins, and WNT-related receptors, while fibroblasts predominantly express extracellular matrix components^75^(Fig. 4d). In the steady state, interactions with fibroblasts are dominated by homeostatic ECM components, including basement membrane. Activation introduces integrin-mediated interactions between LCs and fibroblasts (e.g., *Itga9_Itgb1*) and immune-related signaling (innate immune, chemokine, cytokine), reflecting ECM remodeling and immune readiness. In the migratory state, LCs shift ECM interactions from *Cd44* to high-affinity^76^ integrins (*Itga9_Itgb1, Itga5_Itgb1, Itgav_Itgb1*). For myofibroblasts, the main interactions are mediated by Notch signaling and adrenergic signaling, which expand as LCs are activated and then migrate (Fig. 4d). Taken together, these progressive changes in LC-fibroblast interactions reflect a coordinated remodeling of the cell communication landscape, enabling the transition of LCs from tissue-resident maintenance to active immune engagement and migration.

### Divergent migratory mechanisms of LCs and dermal dendritic cells

In addition to cell–cell communication, we observed a notable overlap between migratory state LCs and migratory dendritic cells (DCs)^77^, converging into a single cluster (Fig. 4e). LCs are known to exhibit features of both macrophages and dendritic cells. However, it remains unclear whether these characteristics shift depending on the activation state of LCs. Surprisingly, among all LC states, the migratory population exhibits the greatest transcriptional similarity to migratory DCs. This includes genes associated with antigen presentation, immune activation, and lymph node homing, such as *Ccr7, Cd80, Cd86*, and *Irf8*. Interestingly, despite this convergence, they still retain distinct molecular features reflective of their origins and functions. Migratory DCs exhibit a strong interferon-stimulated gene signature, expressing genes such as *Ifit1, Rsad2*, and *Oasl2*, indicating a heightened antiviral and pro-inflammatory profile. In contrast, migratory state LCs preserve epidermal and tissue-resident characteristics, marked by elevated expression of *Aldh1a2, Pparg*, and *Rora*, and produce regulatory cytokines such as *Il10* and *Il23a*, suggestive of an immune-modulatory role with potential Th17/Th2 skewing. Additionally, LCs express *S1pr1* and *S1pr3*, implicating sphingosine-1-phosphate signaling in their tissue egress^78,79^, in contrast to DCs, which predominantly utilize the chemokine axis *Ccr7–Ccl19/21*^80^ (Fig. 4e). Collectively, these findings highlight a shared immunogenic program between LCs and DCs, while revealing distinct molecular features and divergent migration strategies.

### Activated state LCs potentiate antigen-driven adaptive immune responses

**Fig. 5.**
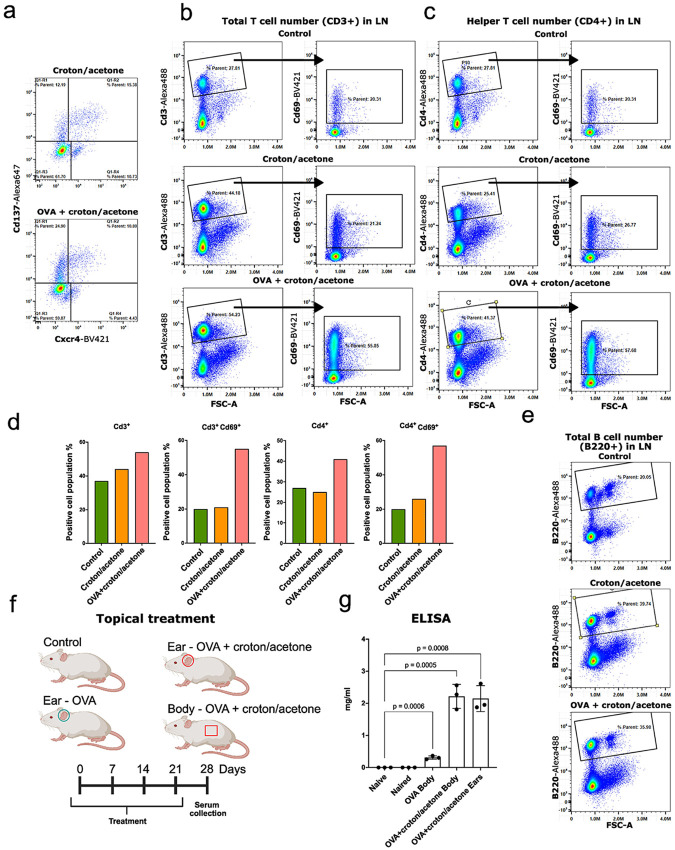
Simultaneous application of OVA and croton oil enhances immune activation. **a.** The number of activated LCs increases significantly under combined treatment. Flow cytometry is representative data (n = 3 mice). **b.** A higher number of activated CD3^+^CD69^+^ T cells is observed in the lymph nodes. Flow cytometry is representative data (n = 3 mice). **c.** Phenotypic analysis confirms that the activated T cells are predominantly T helper cells. Flow cytometry is representative data (n = 3 mice). **d.** Bar plot quantifying the total number of T cells across treatment conditions (n = 3 mice). **e.** The number of B cells also increases in response to combined OVA and croton oil treatment. Flow cytometry is representative data (n = 3 mice). **f.** Schematic illustrating the timeline and treatment conditions for topical application of OVA and/or croton oil. **g.** Co-application of croton oil and OVA leads to robust production of anti-OVA IgG antibodies, in case of body treated 2 x 2 cm^2^, indicating a strong humoral immune response (n = 3 mice).

As described above, activated state LCs express genes associated with phagocytosis and proteasome-mediated antigen degradation, key processes in immunological antigen presentation. To determine how the extent of LC activation differs under antigen exposure, we compared responses to croton/acetone treatment alone versus the same treatment with the addition of excess antigen, specifically ovalbumin (OVA)^81^. For this experiment, we established multiple groups of mice receiving different treatments (Fig. 5a): one group received topical application of OVA, the second was treated with croton/acetone alone, and the third received a combination of OVA and croton/acetone. After 48 hours, flow cytometric analysis revealed a higher number of Cd137^+^ activated state LCs in the group treated with combined OVA and croton/acetone (Fig. 5b-d). This observation suggests that excess antigen enhances activation of LCs, leading to an increased proportion of activated-state cells.^82^. To further investigate the immune response, we isolated lymph node cells from all groups. In mice that received OVA and croton/acetone, there was a significant increase in Cd69^+^ T helper cells, indicating early T cell activation (Fig. 5b-d). Additionally, staining for B220 (*Cd45r*) reveals increased B cells in this sample, suggesting an augmented humoral immune response as well ^83^ (Fig. 5e).

To functionally assess the humoral immune response, we quantified antibody production. We divided mice into four experimental groups and administered different combinations of OVA and croton/acetone: untreated, ears treated with OVA only, ears treated with OVA and croton/acetone, and 2 x 2 cm^2^ body skin treated with OVA and croton/acetone (Fig. 5f). Treatments were administered once every 7 days for one month, at which time blood was collected and serum was isolated. The presence of IgG antibodies to OVA was analyzed using ELISA. We find that mice treated with OVA and croton/acetone have a higher amount of anti-OVA IgG antibodies (Fig. 5g). This result highlights the functional relevance of the activated LC state in promoting systemic humoral immunity.

Altogether, these findings demonstrate that the activated state of LCs is not merely a transient intermediate, but a functionally distinct state with enhanced capacity for immune priming. In addition, antigen exposure during this state significantly amplifies downstream adaptive immune responses, including both T cell activation and antibody production.

### The complement cascade coordinates local immune responses by attracting LCs to wounded areas

**Fig. 6.**
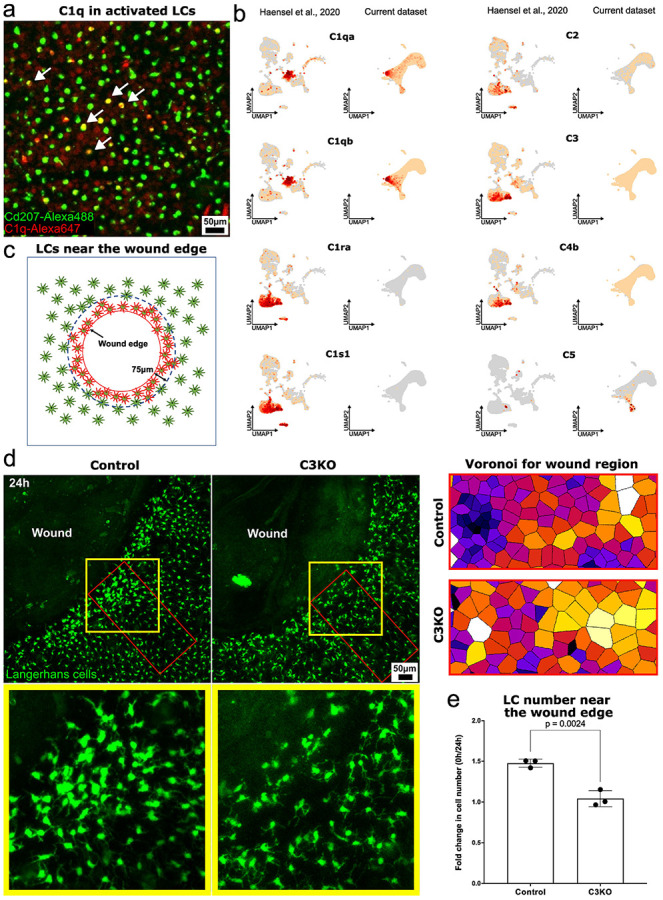
Role of complement signaling in LCs response to skin lnjury. **a.** A representative image of C1q expression in activated LCs 48 hours after croton/acetone treatment of ear skin; red – C1q, green – Lang-GFP (n = 3 mice). **b.** Distinct epidermal cell types express different components of the complement system, indicating cell type–specific roles in complement activation and regulation. **c.** Schematic representation of the 75 μm zone surrounding the wound. **d.** Representative images and Voronoi diagrams of LCs near the wound edge from control and C3 KO mice. Knockout of the central complement component C3 impairs the recruitment of activated LCs to the wound edge, highlighting the essential role of C3 in LC-mediated immune surveillance during tissue injury (n = 3 mice). **e.** Comparison of LC enrichment within a 75 μm circumferential zone around the wound in control and C3KO mice. Cell fold change at 48 hours post-injury were compared with those immediately after wound creation in the same mouse. Bar plot statistics were calculated using a paired t-test (n = 3 mice).

To better understand the functional relevance of the activated LC state, we examined upstream regulatory pathways that may orchestrate its initiation. Our transcriptomic analysis of LCs implicates the complement system as a key innate immune pathway involved in LC regulation (Fig. 3b). In particular, activated state LCs express C1q subunits, which are central to initiating the classical complement pathway (Fig. 6a) ^84^. At the same time, fibroblasts, especially the WNT-modulated subset^85^, express *C1s1* and *C1ra*, serine proteases required for the activation of C1q (Fig. 6b). This interaction supports the initiation of the complement cascade, promoting pathogen recognition and clearance through opsonization^86^. Furthermore, WNT-modulated fibroblasts express *C3*, a central component of the complement system, along with *C4b*, which is required for the conversion steps in the complement cascade^87^(Fig. 6b). This expression pattern highlights the cooperative interaction between LCs and fibroblasts in coordinating a local immune response.

One *C3* derivative is the anaphylatoxin C3a, known to attract antigen presenting cells to sites of inflammation. It has never been described in the context of LC function despite the fact that LCs are known to express an anaphylatoxin receptor^88^. To investigate the functional role of *C3* in complement activation and LC localization, we generated *C3*-deficient (C3KO) mice combined with the *Lang-GFP* allele to visualize LCs with intravital imaging. We created 1 mm diameter circular wounds on the ears of both C3KO and control (only expressing Lang-EGFP) mice and used intravital microscopy to quantify LCs within 75 μm of the wound edge (Fig. 6c). In control mice, LCs lost their regular distribution and became increasingly localized near the wound edge 24 hours after injury. In contrast, C3KO mice showed little change in LC localization near the wound, despite clear signs of LC activation and spatial disorganization. Together, our results reveal that the complement system plays a critical role in initiating LC activation during early injury responses. This process is supported by fibroblast-derived complement components, underscoring a cooperative interaction between LCs and stromal cells.

In conclusion, we show that the activated state of LCs is not merely defined by transcriptional priming for antigen presentation, but also reflects active crosstalk between LCs and diverse cell populations within the damaged or inflamed epidermis. Notably, not all these interactions are detectable through transcriptome analysis alone, underscoring the need for high-quality data acquisition and integrative approaches to fully elucidate intercellular communication in inflamed skin. Our findings expand the traditional view of LCs from passive antigen sentinels to dynamic regulators of immune coordination and tissue repair.

## Discussion

LCs are tissue-resident immune cells in the epidermis that play an important role as a first-line immune barrier against the external environment. Upon activation by diverse external stimuli, LCs migrate to the lymphatics and initiate subsequent adaptive immune responses. However, transcriptomic dynamics and functional consequences of LC activation are poorly understood. Applying scRNA-seq to murine epidermal LCs afforded us an unprecedented opportunity to explore LC transcriptional diversity and functional specialization. Here, we obtained a high-quality single-cell dataset of over 22,000 LCs encompassing the entire activation trajectory. Our data capture the dynamic progression of LCs from the homeostatic steady state through an intermediate activated state to the fully activated migratory state. The intermediate activated state was previously unknown, and its presence provides clues to how and why LCs shift from local immune surveillance to orchestrating broader immune responses and tissue repair.

scRNA-seq analysis combined with RNA velocity measurement reveals that LCs exhibit a common activation trajectory in response to both physical (stamp-punch) and chemical (croton/acetone) stimuli, indicating that LCs respond similarly to distinct stimuli. Using intravital imaging, we confirmed that LCs undergo similar spatial redistribution at comparable time points following both treatments. This allowed us to isolate and characterize activated state LCs, a previously unrecognized intermediate population. We assembled a detailed map of the molecular features of the activated state, separate from those of the migratory and steady states. Activated state LCs exhibit enhanced glycolytic activity, upregulation of complement system components, and increased expression of phagocytosis-related genes. These changes suggest a metabolic and functional shift that prepares LCs for both local immune catching and subsequent migration to lymphoid tissues. In addition, we show that specific markers such as *Cd137, Ly6a*, and *Cd14* reliably distinguish activated state LCs, providing an unprecedented basis for convenient activated LCs sorting in future studies.

Our analysis reveals that LCs transition through three distinct states — steady, activated, and migratory — with unique transcriptional, metabolic, and immunological features. In the steady state, LCs rely on homeostatic signaling and fatty acid oxidation to maintain immune surveillance. Upon activation, they shift to glycolysis and upregulate genes related to phagocytosis and antigen processing, preparing for immune priming. Migratory LCs require oxidative metabolism and express genes for cytoskeletal remodeling and tissue egress. These coordinated changes suggest that each state is functionally specialized, linking cellular programs to immune tasks. Further studies are needed to dissect how these state-specific features influence LC behavior and immune outcomes in vivo.

Notably, our findings highlight the complement system as a key regulator of LC behavior during skin inflammation. The spatially coordinated expression of complement components, where fibroblasts serve as local sources of C3 and its activating enzymes, and LCs express complement receptors. This result suggests a fibroblast–LC signaling axis that links tissue damage to immune activation. The impaired LC recruitment in C3-deficient mice underscores the functional relevance of this axis, indicating that complement signaling may serve as a molecular threshold mechanism to distinguish significant tissue injury from minor, non-inflammatory perturbations.

Furthermore, we find that LCs transitioning to the migratory state begin expressing *C5*, the precursor of the potent anaphylatoxin C5a. This likely promotes recruitment of dermal immune cells to sites where LCs exit the epidermis, implying that LCs not only initiate adaptive immunity via lymph node migration but also coordinate local innate immune responses during early inflammation. Integration of our dataset with Haensel et al. (2020) revealed no alternative sources of *C5* in wounded skin, suggesting that LCs are a primary source of C5 under pathological conditions. Clinical and *in vivo* evidence links *C5* cleavage products with inflammatory skin diseases such as psoriasis and atopic dermatitis^89-94^. Thus, these findings raise the possibility that dysregulated LC-derived complement activity contributes to pathological inflammation.

In conclusion, we define a distinct activation trajectory of epidermal LCs and uncover a transcriptionally and functionally unique intermediate state characterized by enhanced phagocytic activity and complement engagement. Our findings demonstrate that LCs adopt diverse immune functions depending on their activation state, and our work provides a better understanding of the functional plasticity of LCs during skin injury and immune responses.

## Materials and methods

### Mice

CD-1 mice were obtained from Charles River Laboratories. Lang-EGFP (JAX 016939) and C3 KO (JAX 029661) mice were obtained from were obtained from Jackson Laboratories. K14-H2BmCherry mice were obtained from V. Greco (Yale School of Medicine). To simultaneously visualize LCs, and epithelial cells, Lang-EGFP; K14H2B-mCherry mice were generated. To knock out C3 and visualize LCs, the Lang-EGFP; K14H2B-mCherry; C3 KO^−/−^ mouse was generated. Siblings were used as controls (Lang-EGFP; K14H2B-mCherry; C3 KO^+/−^). Mice were housed in ventilated racks under controlled conditions, including an ambient temperature of 22 °C, relative humidity of 50% ± 10%, and a 12-hour light/dark cycle (lights on from 07:00 to 19:00). Both male and female mice from experimental and control groups were randomly selected for live imaging and LCs isolation. All animals used in this study were between 5 and 8 weeks old. Blinding was not employed. All animal procedures were conducted with approval from the Institutional Animal Care and Use Committee (IACUC) at Michigan State University and conducted in accordance with institutional and national guidelines for animal welfare.

### In vivo LC activation (croton oil/acetone, stamp-punch)

Mice were anesthetized in an isoflurane chamber, and anesthesia was maintained throughout the experiment using vaporized isoflurane delivered via a nose cone, as previously described^95^. Fur was removed from the ears and head using Nair cream (Naircare; 022600003014) applied for 2 minutes using a Q-tip, followed by tap water cleaning and a second 1-minute application. An electric razor was not used to avoid physical damage to the skin. The croton oil solution was freshly prepared before each treatment by mixing croton oil (Sigma; C6719), acetone (Sigma; 179124), and olive oil (Sigma; O1514) in a 4:5:1 ratio. Mouse ears were carefully affixed and flattened onto a plastic cube (15 × 15 mm) using petroleum jelly (Vaseline). Additional petroleum jelly was applied at the base of the ears (proximal to the head) to prevent leakage of the solution. A total of 35 μL of the croton oil mixture was applied to the external surface of each ear and evenly distributed using a plastic pipette tip. Both ears were treated simultaneously. Following application, the mouse remained undisturbed for 10 minutes. The ears were then detached from the plastic cube, and the mouse was returned to the isoflurane chamber for an additional 10 minutes. This procedure was repeated 24 hours later. Croton-oil activated state LCs were isolated 48 hours after the initial treatment. For physical treatment using microneedle puncture, a microneedle array consisting of 38 needles, each 0.2 mm in length (Skinmedix; micro needle dermal stamp 0.2mm; 765573893595), was used. Fur removal and ear fixation were performed as described above. A total of 750 microneedle punctures (neat, without pressing) were applied to each ear, evenly distributed across the ears surface. The procedure was repeated 24 hours later with a reduced number of punctures (150) per ear. LCs from stamp-punched skin were isolated 48 hours after the first treatment.

### Epidermal single-cell suspension preparation and LCs sorting

Mice were treated as described above, while control mice received only Nair treatment at the same time as experimental animals. The procedure was performed 48 hours after the initial treatment in both the croton oil and microneedle punch. Mice were then euthanized following standard protocols after anesthesia with isoflurane. Subsequently, ears were excised, and the skin layer was carefully separated from the underlying cartilage. The isolated skin was placed epidermis-side up in a trypsin solution (0.3% trypsin (Thermofisher; 15090046) in 150 mM NaCl, 0.5 mM KCl and 0.5 mM glucose) and incubated for 1 hour and 45 minutes at 37 °C. Following incubation, the epidermis was gently separated from the dermis using fine forceps. The epidermal sheet was transferred to a scratched-wall plastic Petri dish (Fisher; FB0875711YZ) containing HBSS supplemented with 10% FBS, trypsin inhibitor (Sigma; T6522) and DNaseI (Sigma; D4513), pre-warmed to 37 °C. The epidermis was then mechanically dissociated to a single-cell suspension by pressing it against the scratched surface using forceps, producing a cloudy cell suspension. This suspension was incubated for 15 minutes at 37 °C, then filtered through a 70 μm cell strainer. Dead cells were removed via magnetic separation (Miltenyibiotec; 130-090-101), followed by magnetic enrichment of LCs (Miltenyibiotec; 130-095-408). Cell viability was assessed using either trypan blue staining or flow cytometry (Supplementary protocol 1).

### Single cell library preparation and sequencing

For single-cell RNA sequencing, libraries were prepared according to the manufacturer’s manual using the Chromium Next GEM Single Cell 3' Kit v3.1 (10x Genomics, USA), and sequenced using 2 lanes (2.5GB) NovaSeq X 10B 100 SR (Illumina, USA).

### Single cells data analysis.

#### Preprocessing:

Raw scRNA-seq data were processed using Cell Ranger (v8.0, 10x Genomics) with default parameters and aligned to the GRCm39 reference genome, custom-modified to include additional coding sequences (CDS) for mCherry (AY678264.1) and eGFP (OQ870305.1).

#### Quality Control and Clustering:

Quality control, normalization, clustering, and visualization were performed using Seurat^96^ (v5.2) in R (4.4.0) and Scanpy^97^ (v1.10) with Python 3.10 for RNA Velocity and Cell-cell communication. In our quality control process, we filtered cells based on mitochondrial content and gene/UMI counts using the following Seurat parameters: nFeature_RNA > 1500 & nFeature_RNA < 7000 & nCount_RNA > 1500 & nCount_RNA < 40000 & percent.mt < 3.5. Data were normalized using NormalizeData, clustered using the Louvain algorithm, and visualized via UMAP. Given the LC data homogeneity, UMAP was performed using the top 5 principal components. To avoid integration-related artifacts in our relatively homogeneous dataset and high quality, we initially analyzed LC data without applying advanced integration algorithms. The resulting clustering pattern, including the division into three distinct clusters of LCs, was subsequently validated through integration with the dataset from Haensel et al., 2020.

#### Data Integration:

To integrate with Haensel et al., 2020, we used Seurat’s standard integration workflow. Anchors were identified across datasets using FindIntegrationAnchors, followed by IntegrateData to obtain a corrected expression matrix, which was used for downstream clustering and visualization.

#### Pathway Analysis

SCPA^98^ (v1.6.1) was used to compute single-cell pathway activity scores using curated gene sets (MSigDB^99,100^, Reactome^101^, Wikipathways^102^)

#### Gene Regulatory Networks:

SCENIC^103^ (v0.12.1) was applied to infer transcription factor activity and regulons via GENIE3 (v1.28), RcisTarget (v1.26), and AUCell (v1.28).

#### RNA Velocity:

Velocyto^104^ (v0.17.15) and scVelo^105^ (v 0.2.5) was used for RNA velocity analysis, employing the dynamical model and projecting velocities onto UMAP.

#### Cell–cell Communication:

LIANA++^106^ (v0.1.12) was used to infer ligand–receptor interactions across cell types using consensus rankings from multiple methods.

### Intravital imaging

Mice were anesthetized using an isoflurane induction chamber, and anesthesia was maintained throughout imaging via a nose cone delivering vaporized isoflurane, as previously described^33^. Imaging was performed using Leica TCS SP8 X Two-Photon microscope equipped with a Spectra physics Insight MP Laser and controlled via Leica LAS X software. Z-stack images were acquired using a 25× water-immersion objective (NA 1.0; Leica), with 0.59 × 0.59 mm^2^ field of view. 940 nm wavelength was used for excitation of LCs in Lang-EGFP mice. Z-stacks were acquired in 2 μm steps to cover depths up to 80 μm. For large-area imaging, 9–12 tiled optical sections were acquired using a motorized stage with custom Fiji^107^ (v 1.53) stitching script.

### Voronoi diagram and Clark-Evance index

Spatial distribution of cells was analyzed using Voronoi tessellation in Fiji with the Voronoi plugin. Binary masks of cell centroids were generated, and Voronoi diagrams were computed. The area of each Voronoi cell was measured and used to assess spatial dispersion and local cell density. To quantify the degree of spatial regularity or clustering, the Clark–Evans index (R) was calculated using the Spatstat^108^ package (v 3.3) in R. Cell centroid coordinates were extracted and modeled as a point pattern. The index compares the observed mean nearest-neighbor distance to the expected value under complete spatial randomness (CSR). Values of R > 1 indicate regularity, R < 1 suggest clustering, and R ≈ 1 implies randomness.

### Flow cytometry

For flow cytometric analysis of LC markers, staining was performed prior to the 70 μm filtration step. Draining mandibular lymph node (LNs) were isolated as previously described^109^. LN cell suspensions were prepared using the same procedure as for LCs, excluding the trypsin digestion step. LNs were mechanically dissociated by pressing the tissue against the scratched surface using forceps, producing a cloudy cell suspension. Following preparation of the cell suspension (from epidermis or LNs), the desired number of cells was aliquoted into a 96-well polypropylene v-bottom plate (Corning; 3344). Dead cells were stained using Live/Dead Blue dye (Thermofisher; L34961; 1:800) for 15 minutes at room temperature, followed by two washes with staining buffer. To block nonspecific binding, Fc receptors were blocked using Fc block reagent (BDbiosciences; 553142) for 15 minutes. Next, cells were incubated with nonconjugated, fluorochrome or biotin-conjugated primary antibodies (1:200 dilution) for 30 minutes in the dark (Biolegend: 108103, 123305, 104703, 146511, 100212, 100425, 104545, 103228). After two additional washes with staining buffer, cells were incubated with either streptavidin-conjugated fluorochrome (1:800 dilution) or appropriate fluorochrome-conjugated secondary antibodies (Biolegend: 405237). Following staining, cells were washed several times with staining buffer. Cells were then fixed with 4% paraformaldehyde in PBS for 15 minutes, washed three times with flow cytometry buffer, and subsequently analyzed by flow cytometry. Flow cytometry was performed using the Cytek Aurora spectral flow cytometer. Cells were acquired using SpectroFlo software. Compensation and unmixing were conducted automatically based on single-stain controls using auto fluorescence exclusion function. Data were exported in FCS 3.1 format. Flow cytometry data were analyzed using FCS Express (v 7.24, De Novo Software). Initial gating was performed to exclude debris, doublets, and dead cells. Subsequent gating strategies were applied based on marker expression to identify cell populations of interest. Data visualization included biaxial plots and population statistics.

### OVA treatment

To deliver OVA to LCs, OVA (Invivogen; vac-stova) was diluted in PBS to a concentration of 10 mg/ml. Mice were anesthetized and immobilized; fur was removed from the ear and head, which was then flattened and secured onto a plastic support cube, as described above. A total of 70 μL of 100% absolute ethanol (Sigma; 459836) was applied to each ear surface. Immediately afterward, 30 μL of the OVA-PBS solution was applied and evenly distributed across each ear. The ear with ethanol – OVA mix was then dried for 10 minutes using a stream of argon gas to fix the protein onto the skin surface. After drying, a stable film of OVA formed on the skin surface. Following drying, a mixture of croton oil, acetone, and olive oil (prepared as described above) was applied to the ear and left in place for 10 minutes. Mice were then transferred to the isoflurane chamber an additional 10 minutes. For topical treatment of the body, a 10 × 20 mm bath was formed on the lateral side of the mouse using adhesive tape. The edges of the tape were sealed with petroleum jelly to prevent leakage and ensure localized application. OVA and a mixture of croton oil were applied in the same way as for the ear.

### Whole mount staining

Permeabilized whole-mount staining of full-thickness skin was performed in 24-well plates, following a previously described protocol^110^ using anti-C1q antibody (Abcam: ab182451) and Alexa633 secondary (Invitrogen: A-21070).

### ELISA

Mouse serum was isolated using the gel centrifugation method and then frozen for simultaneous analysis using by standard mouse Anti-OVA IgG enzyme-linked immunosorbent assay (ELISA) following the manufacturer’s protocol (Chondrex; 3011).

### Skin Wound

To create a round wound, we used a punch biopsy tool with a 1 mm diameter circular blade. Mice were anesthetized with isoflurane as previously described. A circular wound was made with a single light motion, carefully avoiding penetration into the dermis. The epidermal layer within the wound area was then gently removed using tweezers.

## Supplementary Material

Supplement 1

Supplement 2

Supplement 3

Supplement 4

Supplement 5 **Supplementary Fig. 1. From data filtering to functional insights. a.** Quantitative and qualitative assessment of data before and after filtering. **b.** UMAP clustering results before and after filtering. **c.** Separate clustering of the untreated sample reveals multiple homeostatic populations of LCs. **d.** An illustration of potential interactions between the complement system and LCs. LCs express specific components such as C1q and C5, while fibroblasts—particularly wound-specific subsets—express C3 along with the enzymes required for the activation and conversion of C1q, C3, and C5.

## Data Availability

All sequencing data generated in this study have been deposited in the Gene Expression Omnibus (GEO) under accession number GSE296148. Additional datasets: filtered and unfiltered data objects, integration and corresponding 10x Loupe browser files are available on Zenodo at https://doi.org/10.5281/zenodo.15319340.
