# Supplementary figures and images for: An intermediate activation state primes Langerhans cell migration from the epidermis
Source: bioRxiv. 2025 Sep 17:2025.05.29.656912. Originally published 2025 May 30. Preprint. [Version 2] doi: 10.1101/2025.05.29.656912 (PMC12154774; doi:10.1101/2025.05.29.656912)

a

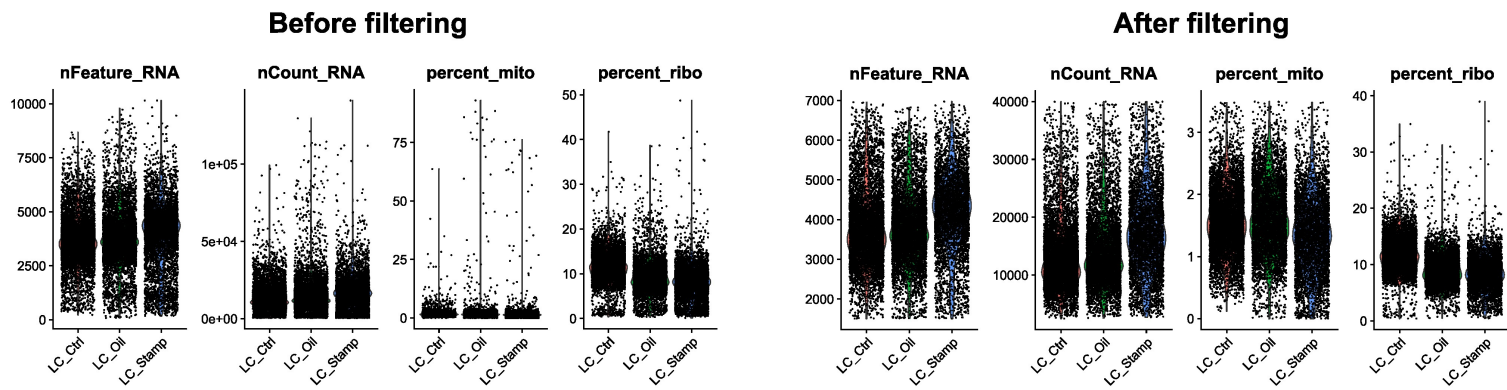

b

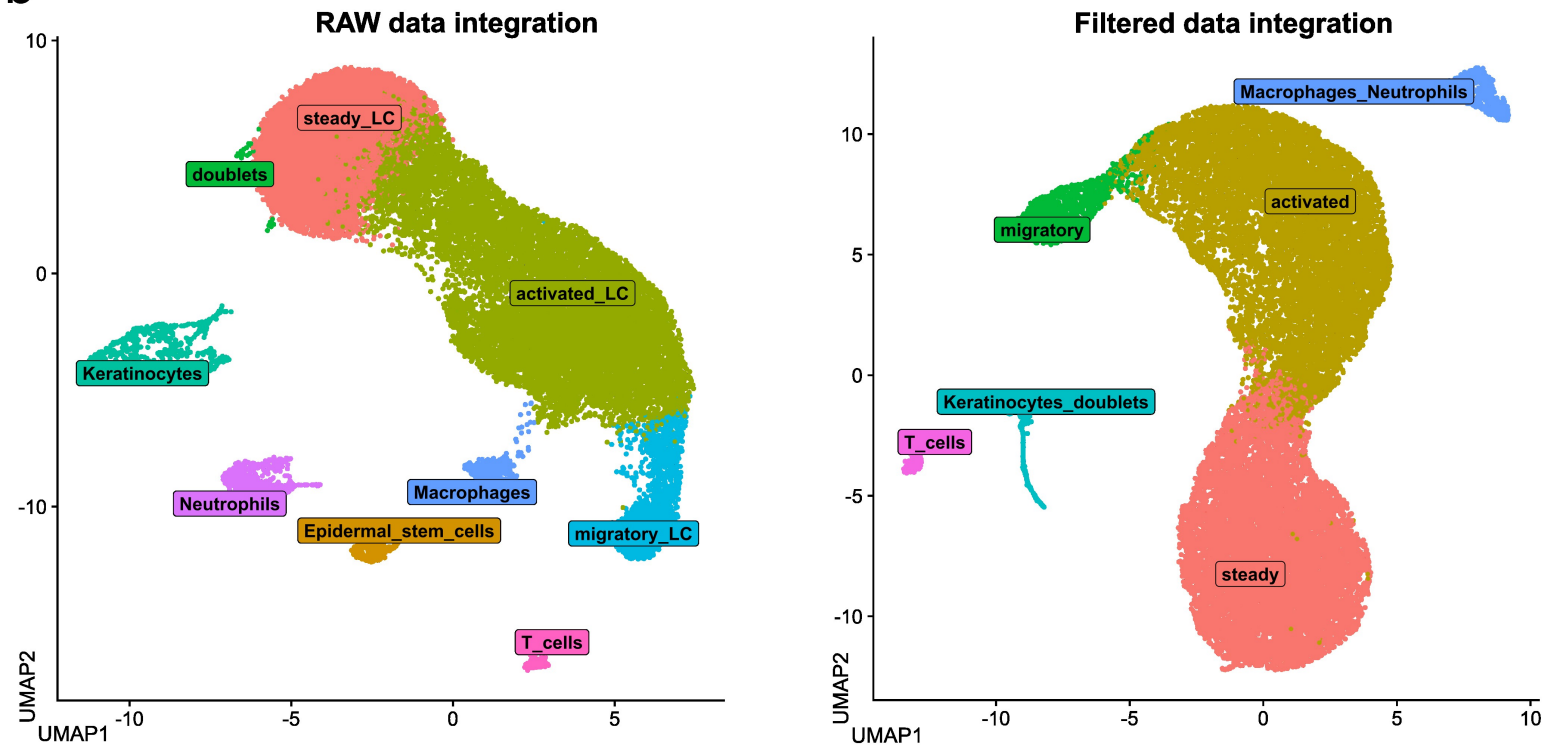

c

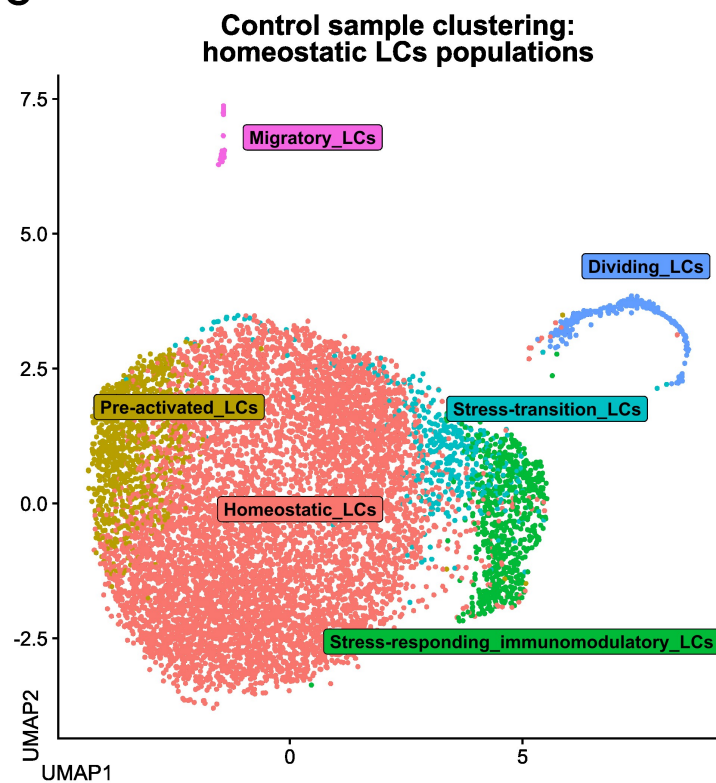

d

### Complement system in LCs activation and attraction

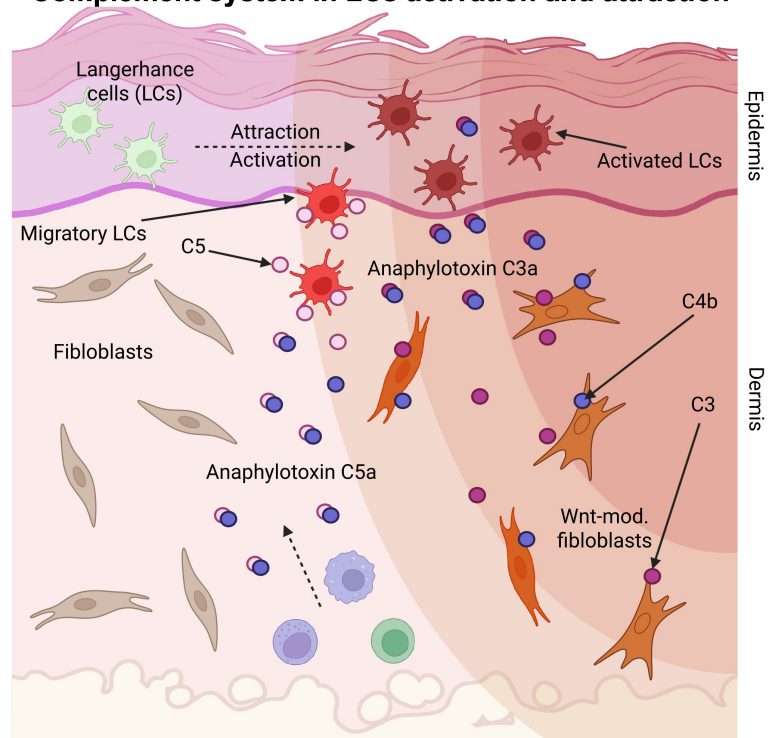

Supplement: Supplement 5 — Supplementary Fig. 1. From data filtering to functional insights. a. Quantitative and qualitative assessment of data before and after filtering. b. UMAP clustering results before and after filtering. c. Separate clustering of the untreated sample reveals multiple homeostatic populations of LCs. d. An illustration of potential interactions between the complement system and LCs. LCs express specific components such as C1q and C5, while fibroblasts—particularly wound-specific subsets—express C3 along with the enzymes required for the activation and conversion of C1q, C3, and C5. [file NIHPP2025.05.29.656912v2-supplement-5.pdf]
